# The therapeutic potential of *Pediococcus pentosaceus* in systemic diseases: current evidence and future prospects

**DOI:** 10.3389/fmicb.2026.1820634

**Published:** 2026-06-30

**Authors:** Wubing Ying, Wenfeng Zhu, Qi Zheng, Jiahao Xie, Guoping Sheng

**Affiliations:** 1 Zhejiang Chinese Medical University, Hangzhou, China; 2 State Key Laboratory for Diagnosis and Treatment of Infectious Diseases, National Clinical Research Center for Infectious Diseases, National Medical Center for Infectious Diseases, Collaborative Innovation Center for Diagnosis and Treatment of Infectious Diseases, The First Affiliated Hospital, Zhejiang University School of Medicine, Hangzhou, China; 3 Key Laboratory of Artificial Organs and Computational Medicine of Zhejiang, Department of Infectious Diseases, Shulan (Hangzhou) Hospital, Shulan International Medical College, Zhejiang Shuren University, Hangzhou, China

**Keywords:** immunomodulation, intestinal barrier function, *Pediococcus pentosaceus*, probiotic properties, probiotics, systemic diseases, therapy

## Abstract

Pediococcus pentosaceus (*P. pentosaceus*) is a lactic acid bacterium widely distributed in fermented foods, dairy products, human specimens, and animal-associated environments. In recent years, *P. pentosaceus* has attracted considerable attention as a potential probiotic candidate due to its intestinal adaptability, antibacterial activity, antioxidant capacity, immunomodulatory effects, and ability to regulate host–microbiota interactions. However, existing findings remain inconsistent, and its systemic therapeutic value has yet to be comprehensively evaluated from the perspectives of strain specificity and translational medicine. This review examines the probiotic characteristics, oral safety, and potential mechanistic applications of *P. pentosaceus* in systemic diseases. Current evidence suggests that different strains may exert beneficial effects through multiple interrelated mechanisms, including enhanced intestinal colonization, production of exopolysaccharides (EPS) and bacteriocins, regulation of short-chain fatty acids (SCFAs), restoration of intestinal barrier integrity, inhibition of inflammatory signaling pathways, modulation of oxidative stress, and alteration of bile acid and other microbial metabolite profiles. These mechanisms are associated with potential benefits across hepatobiliary, gastrointestinal, metabolic, respiratory, neurological, and inflammation-related conditions. However, most current evidence derives from *in vitro* experiments or animal models, clinical evidence remains limited and frequently based on multi-strain formulations, and considerable variability exists across studies in strain origin, dosage, intervention duration, outcome measures, and safety assessment methods. Findings from individual strains should therefore not be extrapolated to the species as a whole. Overall, *P. pentosaceus* represents a promising but insufficiently validated probiotic candidate for adjunctive disease management, and future research should prioritize standardized strain characterization, causal mechanism validation, dose-response assessment, long-term oral safety evaluation, and well-designed single-strain clinical trials.

## 1 Introduction

The human gastrointestinal tract contains trillions of bacteria that engage in both competition and cooperation for restricted space and resources. The structure and dynamics of this microbial population are fundamentally connected to human health (Bäckhed et al., 2005; Sender et al., 2016). Lactic acid bacteria, a prevalent bacterium, possesses potential for adjunctive disease therapy. *Pediococcus pentosaceus* (*P. pentosaceus*), a member of lactic acid bacteria, was initially identified by Claussen during his investigation of “Sarcina disease” in beer and was formally designated by Nakagawa and Kitahara in 1959 (Garvie, 1974; Koutsoumanis et al., 2021). *P. pentosaceus* is classified by the National Center for Biotechnology Information (NCBI) as a Gram-positive coccus within the domain Bacteria, phylum Firmicutes, class Bacilli, order Lactobacillales, family Lactobacillaceae, and genus *Pediococcus*, and is a significant colonizing bacterium in the gut.

**FIGURE 1:**
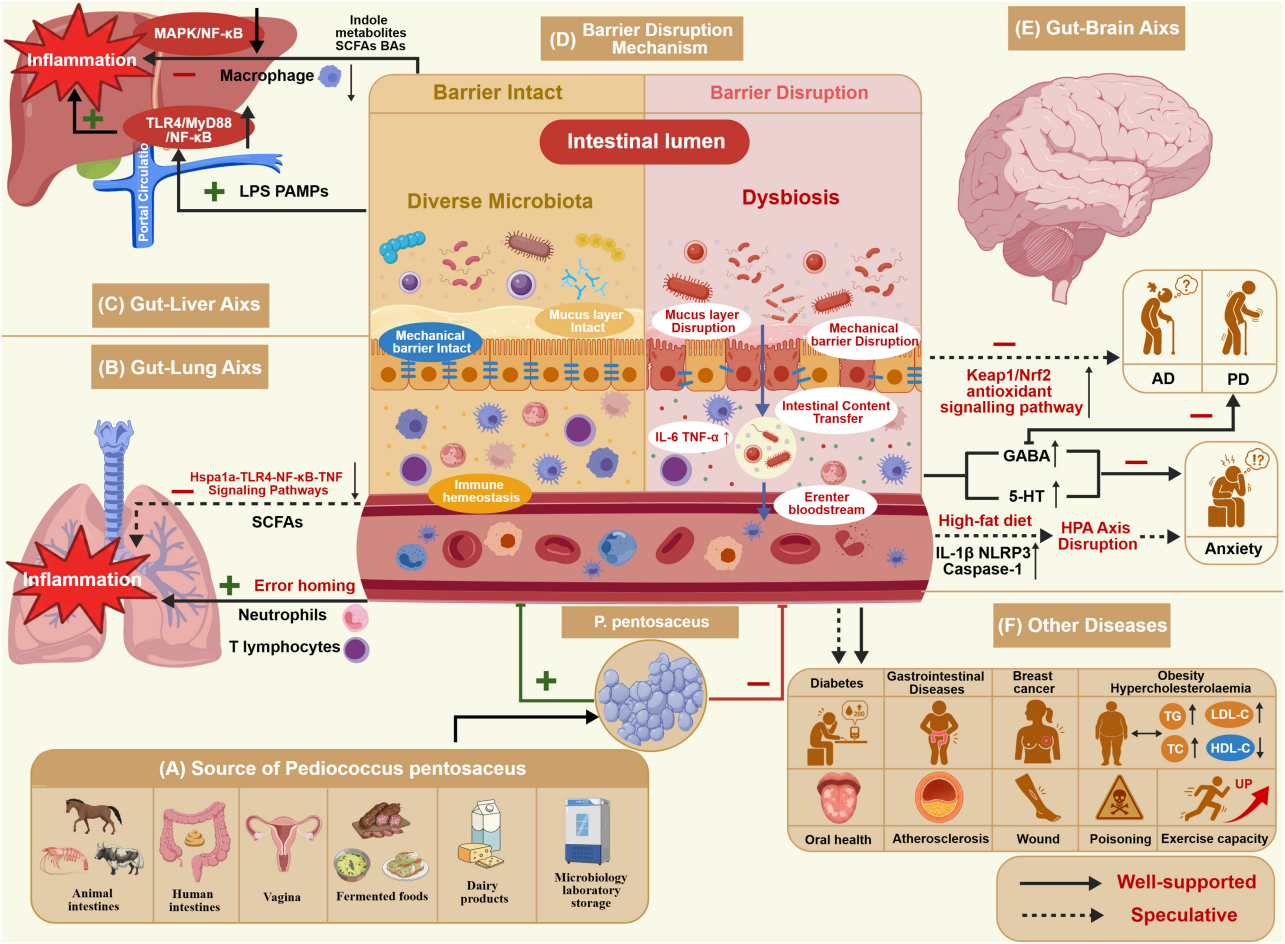
**(A)** Source of *Pediococcus pentosaceus*: *P. pentosaceus* has been isolated from diverse sources, including animal and human intestines, the vaginas of healthy women, fermented foods, dairy products, and microbiology laboratory environments (Well-supported). **(B)** Gut-Lung Axis: (1) Intestinal microbiota dysbiosis impairs the intestinal barrier and increases local pro-inflammatory mediators. This promotes the recruitment of neutrophils and T lymphocytes, which may enter the systemic circulation and aberrantly home to the lungs, thereby inducing pulmonary inflammation (Well-supported). (2) *P. pentosaceus* increases the abundance of SCFAs-producing intestinal bacteria and elevates SCFAs levels. Increased SCFAs inhibit the Hspa1a–TLR4–NF-κB–TNF inflammatory cascade, thereby reducing pulmonary inflammation (Speculative). **(C)** Gut-Liver Aixs: (1) Intestinal LPS and other pathogen-associated molecular patterns (PAMPs) enter the portal circulation and activate hepatic inflammation through the TLR4/MyD88/NF-κB pathway (Well-supported). (2) *P. pentosaceus* produces SCFAs, indole metabolites and regulates bile acid metabolism, thereby inhibiting hepatic macrophage activation and suppressing MAPK/NF-κB signaling, ultimately alleviating hepatic inflammation (Well-supported). **(D)** Barrier Disruption Mechanism: (1) Barrier Intact: The intestinal lumen harbors a diverse microbial community; the mucus layer remains intact; the intestinal epithelial mechanical barrier is preserved; and immune homeostasis is maintained within the lamina propria (Well-supported). (2) Barrier Disruption: Intraluminal microbial dysbiosis occurs, accompanied by thinning of the mucus layer, impairment of the mechanical barrier formed by intestinal epithelial cells, and increased pro-inflammatory factors within the lamina propria. These changes promote the translocation of intestinal contents from the lumen into the circulation, ultimately contributing to systemic inflammation (Well-supported). **(E)** Gut-Brain Aixs: (1) Mitigate neuronal oxidative damage by modulating the Keap1/Nrf2 antioxidant signaling pathway, thereby alleviating Parkinson’s disease (PD)- and Alzheimer’s disease (AD)-related neurodegenerative injury (Speculative). (2) *P. pentosaceus* increases brain GABA levels to alleviate PD-related neurological dysfunction (Well-supported). (3) *P. pentosaceus* increases brain GABA and 5-HT levels to alleviate anxiety-related symptoms (Well-supported). (4) High-fat diet → Increased IL-1β, NLRP3, and Caspase-1 → Hypothalamic–Pituitary–Adrenal (HPA) axis dysfunction → Anxiety (Speculative). **(F)** Other Diseases *P. pentosaceus* also shows potential therapeutic value in other conditions, including diabetes, gastrointestinal disorders, breast cancer, obesity, hyperlipidemia, oral health maintenance, atherosclerosis, trauma, intoxication, and the enhancement of athletic performance. Due to space limitations, only brief examples are provided here; further details are available in the main text and Table 2 (Well-supported/Speculative). Created with BioGDP.com (Jiang S. et al., 2025).

There are several different sources of *P. pentosaceus*, including as dairy products, feces, and fermented foods (Jiang et al., 2021). Research on its potential in the food business and in the treatment of disease has grown in recent years. Numerous studies have demonstrated its therapeutic potential across various conditions, including atherosclerosis (AS) (Kim et al., 2019), colorectal cancer (CRC) (Dubey et al., 2023), diabetes mellitus (DM) (Ratanaburee et al., 2013), Hypercholesterolemia (Damodharan et al., 2015), and constipation (Huang et al., 2020), among others (Figure 1). Research on *P. pentosaceus* has been largely conducted using *in vitro* systems and animal models. Although these studies indicate that the bacterium may confer beneficial effects by modulating intestinal barrier integrity, immune-inflammatory pathways, oxidative stress, and microbial metabolism, supporting evidence remains limited. Most work has focused on individual strains, results vary across disease models, and robust clinical data remain scarce. As such, the clinical potential of *P. pentosaceus* has not been firmly established. Existing reviews have mainly addressed strain selection, food processing, and biopreservation. Far less attention has been paid to its systemic therapeutic value and clinical applicability. This review therefore provides an integrated overview of these understudied areas. We first outline the key biological functions and probiotic properties of *P. pentosaceus*, with an emphasis on its major mechanisms of action and oral safety. We then organize its reported effects by organ system and evaluate the strength of current evidence. Finally, we discuss ongoing challenges and future directions toward clinical translation.

## 2 Materials and methods

This study was designed as a structured narrative review. It aimed to summarize the probiotic properties of *P. pentosaceus*, its oral safety, and its possible therapeutic and translational value in diseases affecting multiple body systems.

### 2.1 Literature search strategy

To gather relevant studies, we searched PubMed, Google Scholar, Web of Science, and Scopus. The search included all records from database inception to March 1, 2026. We used the following main search terms: “*Pediococcus pentosaceus*,” “probiotic,” “gut,” “microbial,” “intestinal barrier,” “inflammation,” “immunomodulation,” “antioxidant,” “bacteriocin,” “safety,” “colitis,” “liver,” “diabetes,” “respiratory,” “neural,” and “cancer.” These terms were searched both alone and in various combinations.

### 2.2 Inclusion and exclusion criteria

We included studies that met the following conditions: (1) original research articles, animal studies, *in vitro* studies, clinical studies, randomized controlled trials, and relevant reviews that investigated *P. pentosaceus* or discussed mechanisms directly relevant to its probiotic functions and disease-related applications; (2) review articles or original studies discussing probiotic functions and their links to specific systemic diseases; (3) articles published in English.

We excluded studies in three situations: (1) the article did not clearly report mechanisms or conclusions related to *P. pentosaceus*; (2) the publication was a conference abstract, editorial, or duplicate report; (3) the full text was unavailable, or the study lacked a sound methodological basis.

### 2.3 Study selection and data extraction

After applying the inclusion criteria, we read the full text of eligible studies. From each paper, we extracted key information, including strain number, source, study design, disease model, major probiotic features, proposed or confirmed mechanisms, therapeutic effects, and safety findings. When disagreements arose during study selection or data interpretation, the authors discussed them until a consensus was reached. After data extraction, we grouped the strains and findings into clear categories. The grouping was based on shared mechanisms, similar disease models, or studies addressing the same topic but reporting different conclusions.

Given that this review was designed as a structured narrative review rather than a systematic review or meta-analysis, a formal risk-of-bias assessment was not performed. Instead, we developed a simplified evidence-support classification informed by the general principles of the GRADE, OCEBM, and JBI frameworks, based on study design, biological plausibility, and the presence or absence of direct mechanistic validation. Findings were accordingly categorized into three levels: “High Support,” “Moderate Support,” and “Low Support.” Findings supported by strain-specific clinical evidence or by *in vivo* studies with direct mechanistic validation were classified as “High Support.” Those supported by *in vivo* evidence lacking definitive causal validation, or by clinical studies using multi-strain probiotic formulations that precluded strain-specific attribution, were classified as “Moderate Support.” Findings based primarily on *in vitro* experiments, genomic predictions, microbiome or metabolite correlation analyses, or indirect mechanistic inference were classified as “Low Support.” This classification was intended to summarize the strength of mechanistic support for each finding, rather than to provide formal clinical recommendations (Guyatt et al., 2008; Howick et al., 2011; The Joanna Briggs Institute Levels of Evidence and Grades of Recommendation Working Party, 2014).

## 3 Probiotic properties of *Pediococcus pentosaceus*

### 3.1 Intestinal colonization

Following oral administration, probiotics must endure the hostile conditions of the digestive system, including gastric acid and bile, to arrive at the intestines. Moreover, the current gut microbiota demonstrates colonization resistance to probiotics, which is intricately linked to resource competition among bacteria (Han et al., 2021). Probiotics can enhance intestine colonization by secreting different chemicals, primarily found on the cell surface or inside the extracellular matrix of the probiotics. These compounds, by interacting with host mucus, extracellular matrix proteins, or by establishing cross-links with extracellular polysaccharide matrices, collaboratively facilitate the adhesion, colonization, and biofilm formation of probiotics on host tissues (Fischetti, 2016; Hymes and Klaenhammer, 2016; Singh et al., 2018; Han et al., 2021; Netrusov et al., 2023). Including mucin-binding proteins (MUBs) (Phùng et al., 2024), fibronectin-binding proteins (FBP) (Dhakephalkar et al., 2024), surface layer proteins (SLPs) (Sha et al., 2023), exopolysaccharides (EPS) (Lee M. G. et al., 2022), moonlighting proteins (ENO, GAPDH) (Liu W. et al., 2024), and aggregation-promoting factors (Apf) (Wang et al., 2024).

#### 3.1.1 Surface factors for adhesion

*Pediococcus pentosaceus Li05 (CGMCC 7049)* was extracted from the feces of a healthy individual. Lu et al. (2022) reported that the surface EPS of *P. pentosaceus LI05 (CGMCC 7049)* may help protect the bacterium during gastrointestinal transit. Under bile salt conditions, the EPS layer appears to undergo structural changes, which may increase interactions with mucin and possibly expose adhesive structures such as pili, thereby enhancing intestinal adhesion (Lu et al., 2022). Han et al. (2025) employed a biotin–streptavidin bridging strategy to conjugate anti-MUC2 antibodies onto the surface of *P. pentosaceus Li05*, constructing an engineered strain designated *M-Li05* with MUC2-targeting adhesion capabilities. The surface-conjugated anti-MUC2 antibody functions as an artificial adhesin–analogous to natural bacterial mucin-binding proteins (MUBs)–specifically recognizing and binding to MUC2 mucin secreted by goblet cells within the intestinal mucus layer. By increasing the number of MUC2-binding sites on the bacterial surface, this modification enhanced Li05 adhesion to the intestinal mucus layer, thereby facilitating early intestinal colonization (Han et al., 2025). The genome of *P. pentosaceus E3* encodes many adhesion-related factors, including a fibronectin-binding protein (FBP), which may facilitate adherence to fibronectin on intestinal epithelial surfaces. Additionally, the E3 strain encodes a group of cell wall-anchored proteins containing the LPXTG motif (Leu–Pro–X–Thr–Gly, where X represents any amino acid). Sortase recognizes the conserved LPXTG motif, cleaves the threonine (Thr)–glycine (Gly) peptide bond, and covalently anchors the protein to the peptidoglycan layer of the Gram-positive bacterial cell wall. This stable surface display may facilitate bacterial adhesion to mucus, extracellular matrix components, and intestinal epithelial surfaces (Zaghloul and Halfawy, 2024).

#### 3.1.2 Auto-aggregation and hydrophobicity

*Pediococcus pentosaceus I44* was extracted from ileal and cecal biopsy specimens of healthy subjects. *In vitro* tests revealed that the strain displayed significant auto-aggregation, achieving 87% after 24 h, which was further augmented by the incorporation of oleic acid. Auto-aggregation is typically seen as advantageous for intestinal colonization, since it facilitates the formation of micro-aggregates among cells of the same strain, enhances local cell density, and fosters closer interaction with the intestinal mucus layer and epithelial surface. These attributes may facilitate the strain’s establishment of a more stable local microbial barrier and diminish competition from pathogens for attachment sites, hence enhancing its persistence in the gut (Kos et al., 2003; Gómez et al., 2016; Vasudevan et al., 2021). *P. pentosaceus* strain *OBK05*, isolated from buttermilk, exhibits strong auto-aggregation and high cell surface hydrophobicity. Increased cell surface hydrophobicity promotes non-specific interactions between bacterial and host surfaces, mainly via hydrophobic interactions, van der Waals forces, and certain electrostatic contacts. This not only improves bacterial adhesion to the intestinal mucus and epithelial surfaces but also strengthens cell–cell interactions, thereby promoting auto-aggregation (Bhukya and Bhukya, 2021).

#### 3.1.3 Co-aggregation and pathogen inhibition

*Pediococcus pentosaceus* 1101 may exert part of its antibacterial activity through the production of an N-acetylmuramyl-L-alanine amidase, an enzyme that hydrolyzes peptidoglycan and disrupts bacterial cell wall integrity, thereby contributing to the inhibition of susceptible bacteria; however, the mechanism by which this enzyme avoids autodegradation remains unclear given that peptidoglycan is also a key component of the cell wall in strain 1101. Furthermore, *P. pentosaceus* 1101 has been shown to co-aggregate with specific pathogens such as *Listeria monocytogenes*, a cell–cell interaction that may promote the formation of probiotic–pathogen complexes, impede pathogen adhesion to host epithelial surfaces, and potentially reduce pathogen colonization. Collectively, these traits suggest that *P. pentosaceus 1101* possesses an enhanced ability to compete in the intestinal environment and support its persistence in the gut (Escobar-Sánchez et al., 2022). Findings from strains *I44, OBK05* and *1101* suggest that high auto-aggregation, hydrophobicity and co-aggregate supports intestinal colonization, although further confirmation through direct adhesion assays and *in vivo* experiments is still required.

#### 3.1.4 Summary

**FIGURE 2:**
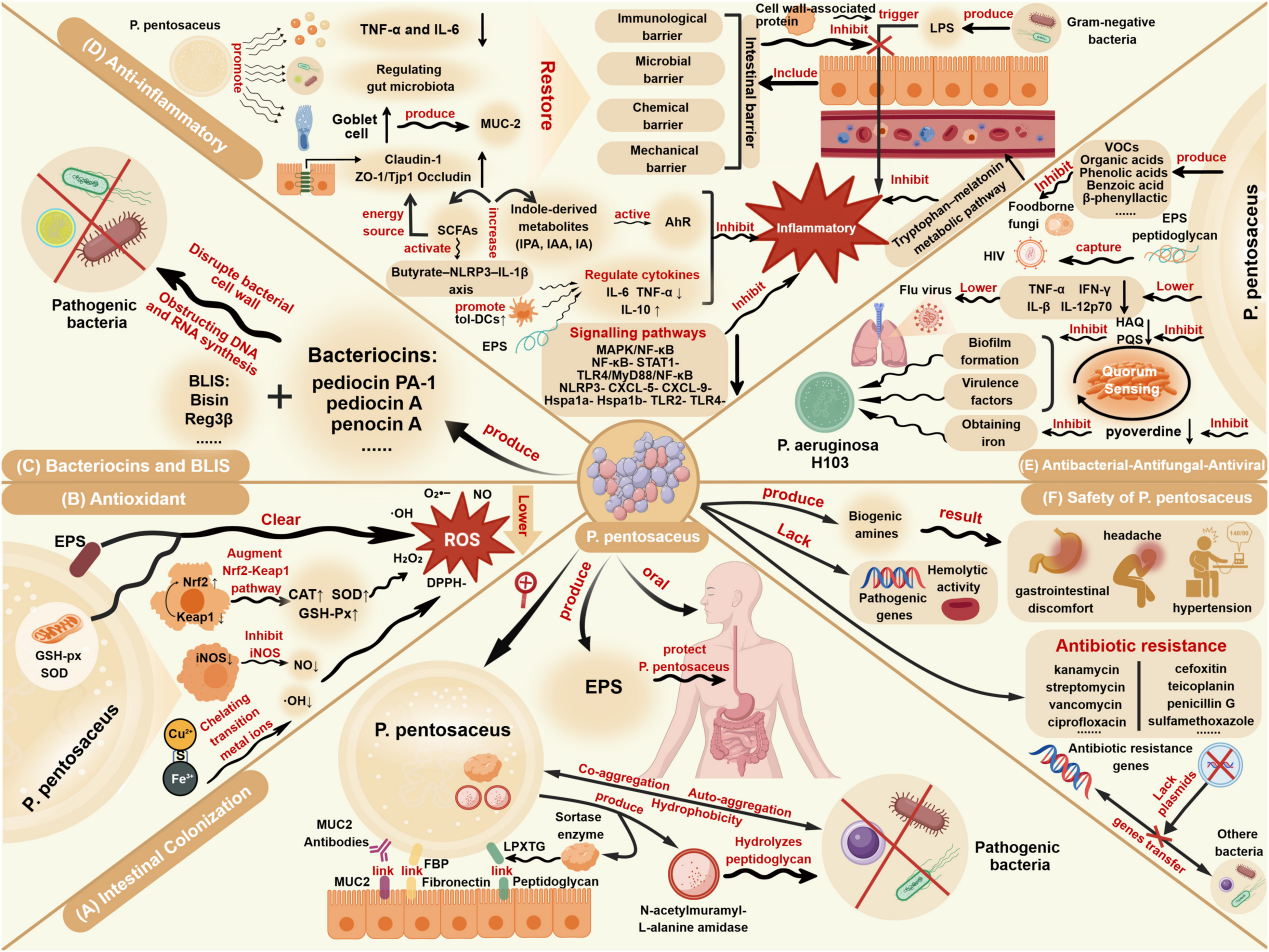
**(A)** Intestinal colonization surface EPS protects the strain from damage during gastrointestinal transit. Surface-bound MUC2 antibodies facilitate adhesion to intestinal mucosal cells expressing the MUC2 protein. Surface FBP mediates binding to fibronectin on the intestinal epithelium. The surface LPXTG motif–following modification by strain-produced Sortase–binds to peptidoglycan on the intestinal mucosal surface. The strain produces N-acetylmuramyl-L-alanine amidase, which hydrolyses the surface peptidoglycan of pathogenic bacteria, thereby killing target organisms. Additionally, auto-aggregation, co-aggregation, and surface hydrophobicity contribute to pathogen inhibition. **(B)** Antioxidant utilizes cell wall-associated EPS to directly scavenge ROS. Enhances intracellular SOD and GSH-Px activity, boosting ROS-scavenging capacity. Activates the Nrf2–Keap1 antioxidant signaling pathway in hepatocytes. Inhibits inducible nitric oxide synthase (iNOS) expression. Chelates transition iron and copper ions to alleviate oxidative stress. **(C)** Antimicrobial effects produces bacteriocins (pediocin PA-1, pediocin A, penocin A) and BLIS (e.g., bisin) to disrupt bacterial cell walls and obstruct DNA and RNA synthesis in pathogenic bacteria. **(D)** Barrier restoration and immunoregulation contains cell wall-associated proteins which attenuates LPS toxicity by promoting the release of fatty acids–including hydroxymyristic acid, palmitic acid, and stearic acid–as well as glucose from LPS. Downregulates TNF-α and IL-6, restoring the intestinal immune barrier. Modulates gut microbiota to restore the intestinal microbial barrier. Upregulates goblet cells to promote MUC-2 secretion, restoring the intestinal mucus barrier. Directly or via increased SCFAs, upregulates Occludin, Claudin-1, and ZO-1 to restore intestinal mechanical barrier integrity. Restores intestinal indole metabolites (IPA, IAA, and IA), activating AhR to alleviate systemic inflammation. Via EPS or by promoting tol-DC differentiation, modulates inflammatory factors to mitigate systemic inflammation. Activates the butyrate–NLRP3–IL-1β axis via increased SCFAs, enhancing local anti-inflammatory capacity. Suppresses systemic inflammation by inhibiting MAPK/NF-κB, TLR4/MyD88/NF-κB, NF-κ B-, STAT1-, Hspa1a/b–TLR2/4–M1 axis-, NLRP3-associated inflammatory signaling, and the CXCL-5/9 chemokine axis, as well as activating the tryptophan–melatonin metabolic pathway. **(E)** Antibacterial, Antifungal and Antiviral Antibacterial: Inhibits the HAQ/PQS-mediated quorum sensing of *P. aeruginosa H103*, suppressing biofilm formation, virulence factor production, and iron acquisition, thereby reducing its infectious virulence. Antifungal: Produces VOCs, organic acids, phenolic acids, β-phenyllactic acid, and benzoic acid, collectively inhibiting foodborne fungi. Antiviral: Through surface-associated peptidoglycans and EPS that capture HIV-1 and prevent target cell contact; additionally, downregulation of TNF, apoptosis, and NF-κB signaling pathways in lung tissue reduces pro-inflammatory cytokines (TNF-α, IL-1β, IFN-γ, and IL-12p70) and pulmonary viral load. **(F)** Safety of *Pediococcus pentosaceus* certain strains are capable of producing biogenic amines, which may trigger headaches, hypertension, and gastrointestinal discomfort. The majority of evaluated strains lack virulence genes and haemolytic activity. Although antibiotic resistance has been observed phenotypically, resistance genes are absent at the genomic level in some strains, while in others they are chromosomally encoded and therefore non-transferable. Only plasmid-borne resistance genes carry the potential for horizontal transfer. Created with BioGDP.com (Jiang S. et al., 2025).

In summary, these findings suggest that *P. pentosaceus* may enhance intestinal colonization through multiple surface-associated traits. However, as much of the current evidence is based on genomic analysis or *in vitro* observations, further *in vivo* studies are still needed to confirm their actual roles in gut colonization. The summary of strains and evidence grading for this part are presented in Table 1. The mechanistic diagram is shown in Figure 2A.

**TABLE 1:** Probiotic properties of *Pediococcus pentosaceus*.

Strain	Nation	Source	Probiotic properties	Year	Mechanism	Study type	Direct experimental validation	Evidence-support classification	References
Li05 (CGMCC 7049)	China	Human intestines	3.1 Intestinal colonization	2021	Surface EPS protects the strain during GI transit.	*In vitro* studies	YES	Low	Lu et al., 2022
					Bile salts may remodel EPS structure and expose adhesion sites (e.g., pili), enhancing gut adhesion.		NO	Low	
				2025	A biotin–streptavidin bridging strategy was used to conjugate anti-MUC2 antibodies onto the bacterial surface, enhancing mucus layer adhesion and facilitating early intestinal colonization.	*In vitro* and animal studies	YES	Moderate	Han et al., 2025
E3	Egypt	Shrimp intestines		2024	Encodes FBP and other adhesion-associated factors, suggesting potential binding to intestinal epithelial surfaces.	*In vitro* studies	NO	Low	Zaghloul and Halfawy, 2024
					Encodes LPXTG motif-containing cell wall proteins and sortase, which recognizes the LPXTG motif and cleaves between threonine and glycine to covalently anchor surface proteins to peptidoglycan, enhancing gut adhesion.		NO	Low	
I44	India	Human intestines		2021	Shows high auto-aggregation, further enhanced by oleic acid, supporting gut colonization potential.	*In vitro* studies	NO	Low	Vasudevan et al., 2021
OBK05	India	Buttermilk		2021	Exhibits high auto-aggregation and strong cell surface hydrophobicity, supporting effective gut colonization.	*In vitro* studies	NO	Low	Bhukya and Bhukya, 2021
1101	Mexico	Fermented food		2022	Produces N-acetylmuramyl-L-alanine amidase that disrupts cell wall integrity of susceptible bacteria.	*In vitro* studies	NO	Low	Escobar-Sánchez et al., 2022
					Shows high co-aggregation, supporting gut colonization potential.				
L1	China	Fermented food	3.2 Antioxidant	2025	Antioxidant activity of fermentation supernatant closely linked to EPS production.	*In vitro* studies	NO	Low	Mustafa et al., 2025
R1	China	Fermented food		2021	Increased intracellular SOD and GSH-Px activities, enhancing scavenging capacity against DPPH, OH, and O_2_∙- radicals under H_2_O_2_-induced oxidative stress.	*In vitro* studies	YES	Low	Zhang et al., 2021
SMM914	China	Sow milk		2022	Activates the Nrf2-Keap1 pathway in hepatocytes, upregulates NQO-1, HO-1, SOD1, and CAT expression, increases GSH-Px, SOD, and CAT activities, and reduces MDA levels.	*In vitro* studies	YES	Low	Wang L. et al., 2022
PP04	China	Fermented food		2021	Activates the Nrf2 signaling pathway, downregulates CYP2E1, and reduces ROS production.	*In vitro* studies	YES	Low	Wang Y. et al., 2021
MG9015	Korea	Feline feces		2021	Inhibits LPS-induced iNOS expression and reduces NO production in macrophages.	*In vitro* studies	YES	Low	Kim K. T. et al., 2021
/	Poland	Fermented food		2025	Chelates transition metal ions (iron and copper), reducing OH generation by inhibiting the Fenton reaction.	*In vitro* studies	NO	Low	Łepecka et al., 2025
732	Korea	Fermented food	3.3.1 Bacteriocins	2024	Secreted bacteriocins that killed *Listeria monocytogenes ATCC 15313*.	*In vitro* studies	NO	Low	Choi et al., 2025
E3	Egypt	Shrimp intestines		2024	Encodes pediocin A and penocin A, likely the primary bacteriocins responsible for inhibitory activity against *Listeria monocytogenes* and *Staphylococcus aureus*.	*In vitro* studies	NO	Low	Zaghloul and Halfawy, 2024
ST3633BG	Korea	Silage		2021	Inhibits *Listeria monocytogenes and* vancomycin-resistant *Enterococcus*, likely via pediocin PA-1 secretion.	*In vitro* studies	NO	Low	Fugaban et al., 2022
MT323062	Pakistan	Vaginal discharge	3.3.2 BLIS	2022	Exhibited bactericidal activity against *Enterococcus faecalis*, mediated by BILS.	*In vitro* studies	YES	Low	Shazadi and Arshad, 2022
LBM 18	Brazil	Maize silage		2020	Produces a proteinaceous BLIS with saccharide moiety, hydrophilic and amphipathic properties, inhibiting *L. sakei*, *E. faecium*, *L. innocua*, *L. seeligeri*, and silage-associated fungi.	*In vitro* studies	YES	Low	de Souza de Azevedo et al., 2020
CGMCC 7049	China	Human intestines		2020	Upregulates Reg3β expression → reduces ethanol-induced bacterial genera (*Staphylococcus*, *Oscillospira*, *Allobaculum*) → restores gut microbiota homeostasis.	In animal studies	NO	Moderate	Jiang et al., 2020
KABP041	Spain	/		2024	Produce bisin and BLIS, which inhibit pathogenic bacteria linked to intestinal dysbiosis and exert potential probiotic effects.	*In vitro* studies	NO	Low	Moreno-Villares et al., 2024
AK-23	Japan	Fermented vegetable pickles	3.4 Anti-inflammatory	2017	Harbors a cell wall-associated protein with LPS-binding and hydrophobic-binding domain motifs, attenuating LPS toxicity by promoting the release of fatty acids and glucose from LPS, possibly via hydrolysis of fatty acid–sugar chain bonds.	*In vitro* studies	NO	Low	Asami et al., 2017
4412	India	Fermented *Manilkara zapota* juice		2025	Reduces cell-surface EPS that suppresses pro-inflammatory cytokines (IL-1β, IL-6, TNF-α) while promoting IL-10, thereby exerting anti-inflammatory effects.	*In vitro* studies	YES	Low	Angelin and Kavitha, 2025
PR-1	China	Human intestines		2023	Improves intestinal barrier integrity by upregulating occludin, claudin-1, and ZO-1.	In animal studies	YES	High	Liu et al., 2023
IM96	China	Turnip rape		2021	Increases intestinal goblet cells and MUC-2 secretion, contributing to mucosal barrier restoration.	In animal studies	YES	High	Li et al., 2021
Li05 (CGMCC 7049)	China	Human intestines		2020	Upregulates Tjp1 /ZO-1 and enriches Verrucomicrobiaceae, Verrucomicrobia, and *Akkermansia*, thereby promoting mucosal barrier repair via improved gut microecological homeostasis.	In animal studies	NO	Moderate	Bian et al., 2020
					Reduces pro-inflammatory cytokines (TNF-α and IL-6), thereby attenuating inflammation-induced damage to epithelial tight junctions.				
Li05 (CGMCC 7049)	China	Human intestines		2020	Increases intestinal SCFAs levels. SCFAs may contribute to the restoration of intestinal barrier function.	In animal studies	NO	Moderate	Jiang et al., 2020
YC	China	Fish intestines		2021	Elevates intestinal butyrate levels via gut microbiota remodeling, activating the NLRP3 inflammasome and promoting neutrophil recruitment through the butyrate–NLRP3–IL-1β axis, thereby enhancing host anti-inflammatory capacity.	In animal studies	YES	High	Shan et al., 2021
KCTC 18308P	Korea	Finger millet (*Eleusine coracana*) gruel		2021	Restores intestinal indole metabolites (IPA, IAA, IA) that act as AhR ligands, thereby alleviating systemic inflammation through AhR activation.	In animal studies	NO	Moderate	Yu et al., 2021
KF159	Korea	Kimchi		2024	Promotes differentiation of tolerogenic dendritic cells (tol-DCs) by upregulating ICOS-L, PD-L1, and IDO.	*In vitro* studies	YES	Low	Eom et al., 2024
					Via tol-DCs that directly enhance IL-10 production or stimulate CD4^+^CD25^+^Foxp3^+^ regulatory T cells to secrete IL-10.		NO		
KCTC 18308P	Korea	Finger millet (*Eleusine coracana*) gruel		2021	Suppress the MAPK/NF-κB associated signaling pathways.	In animal studies	YES	High	Yu et al., 2021
SMM914	China	Sow milk		2024	Suppresses Hspa1a-, Hspa1b-, TLR2-, and TLR4-associated signaling → reduces M1 macrophage polarization.	In animal studies	NO	Moderate	Liu Y. et al., 2024
					Activates tryptophan–melatonin metabolic pathway → enhances endogenous antioxidant metabolism.				
KFT18	Korea	Kimchi		2022	Suppresses NF-κB- and STAT1- associated signaling pathways.	In animal studies	YES	High	Lee J. H. et al., 2022
PP04	China	Fermented food		2021	Suppresses TLR4/MyD88/NF-κB associated signaling pathways.	In animal studies	YES	High	Wang Y. et al., 2021
PP34	China	*Bos grunniens*		2024	Suppresses NLRP3-, CXCL- 5-, and CXCL-9- associated signaling pathways.	In animal studies	YES	High	He et al., 2025
2-5	China	Chicken intestines	3.5.1 Antibacterial	2022	Produces CFS that inhibits *E. coli*, *S. enterica* Typhimurium, and *S. aureus*; likely mediated by organic acids and other extracellular substances.	*In vitro* studies	NO	Low	Wang P. et al., 2022
					Inhibits *S*. Typhimurium adhesion to Caco-2 cells (competition and exclusion assays), possibly associated with its relatively strong epithelial adhesion capacity.				
MZF16	France	Ossban		2025	Attenuates *P. aeruginosa* H103 quorum sensing via reduced HAQ production, weakening its pathogenic potential.	*In vitro* studies	YES	Low	Boujnane et al., 2025
					Reduces pyoverdine production, limiting *P. aeruginosa* H103 access to host-derived iron.		NO		
YC	China	Fish intestines		2021	Enhances host resistance to *Aeromonas hydrophila* infection through the butyrate–NLRP3–IL-1β axis	In animal studies	YES	High	Shan et al., 2021
C15	Spain	Fermented food	3.5.2 Antifungal	2023	Inhibits mycotoxin-producing molds associated with meat spoilage (e.g., *Aspergillus* and *Penicillium*), attributed to the production of organic acids, phenolic acids, and VOCs.	*In vitro* studies	YES	Low	Nazareth et al., 2023
ST3522BG	Korea	Silage		2021	Exhibits *in vitro* inhibitory activity against several food- and feed-associated molds, through the combined action of lactic acid and other metabolites.	*In vitro* studies	YES	Low	Fugaban et al., 2022
M9MM5b S11sMM1 S1M4	Spain	Chicha		2022	Exhibits temperature-dependent antifungal activity against toxigenic *Fusarium* spp., with inhibitory rates of 56.1%–100% at 30 °C in dual-culture assays.	*In vitro* studies	YES	Low	Mateo et al., 2023
Te010	Malaysia	Fermented food		2011	Produces heat-stable antifungal metabolites that delay fungal spoilage in bread.	*In vitro* studies	YES	Low	Muhialdin et al., 2011
VM95 VM21	Spain USA	Human milk	3.5.3 Antiviral	2010	Inhibits R5-tropic HIV-1 infection *in vitro* (49.0% and 45.5%), potentially mediated by surface-associated peptidoglycans and EPS that capture or sequester virions, preventing target cell contact.	*In vitro* studies	NO	Low	Martín et al., 2010
MIANGUAN2 (CGMCC No. 29410)	China	Human intestines		2024	Increases intestinal SCFAs, modulating the gut–lung axis and downregulating TNF, apoptosis, and NF-κB pathways, thereby reducing TNF-α, IL-1β, IFN-γ, and IL-12p70.	In animal studies	YES	High	Chen et al., 2024
					SCFAs levels negatively correlated with pulmonary viral load.				
BalaMMB-P3	Korea	Milk coagulant	3.6.1 Hemolytic activity	2022	Non-hemolytic	*In vitro* studies	YES	Low	Park et al., 2022
CACC616	Korea	Pig intestines		2023		In animal studies	YES	High	Park et al., 2023
L1	China	Fermented food		2025		*In vitro* studies	YES	Low	Mustafa et al., 2025
732	Korea	Fermented food		2024		*In vitro* studies	YES	Low	Choi et al., 2025
OBK05	India	Buttermilk	3.6.2 Antibiotic resistance and the presence of antibiotic-resistance genes	2021	Resistance to kanamycin, streptomycin, vancomycin, ciprofloxacin, and norfloxacin is chromosomally encoded (non-transferable); trimethoprim resistance is plasmid-encoded and potentially transferable.	*In vitro* studies	YES	Low	Bhukya and Bhukya, 2021
E3	Egypt	Shrimp intestines		2024	Exhibits phenotypic resistance to teicoplanin, penicillin G, cefoxitin, sulfamethoxazole/trimethoprim, and colistin sulfate; however, genomic analysis revealed no clinically relevant resistance genes or plasmid replication elements.	*In vitro* studies	YES	Low	Zaghloul and Halfawy, 2024
DSM 20280, DSM 20281, DSM 20336T, LMG 9445, FAM 18048, FAM 18327, FAM 18523	Switzerland	Multiple sources		2021	MIC values for tetracycline and chloramphenicol exceeded recommended cutoffs; however, whole-genome screening revealed no known antibiotic resistance genes across all 35 strains tested.	*In vitro* studies	YES	Low	Shani et al., 2021
KUH5 KUH6 KUH7	Denmark	Cheese	3.6.3 Toxin production potential and the presence of pathogenic genes	2019	Produces histamine *in vitro*, suggesting a potential contribution to biogenic amine accumulation in aged cheese.	*In vitro* studies	YES	Low	Møller et al., 2020
K-B21 K-B01	Korea	Kisra		2024	Completely inhibits tyramine and cadaverine formation during kisra fermentation, while markedly reducing putrescine, histamine, spermine, and spermidine levels.	*In vitro* studies	YES	Low	Hassan et al., 2024
BBS1	China Philippines	Fermented Bamboo Shoots		2025	Harbors genes encoding β-glucosidase (EC 3.2.1.21) and 6-phospho-β-glucosidase (EC 3.2.1.86); β-glucosidase (linamarase) hydrolyses cyanogenic glycosides, contributing to cyanide detoxification during fermentation, consistent with demonstrated linamarase activity and cyanide tolerance.	*In vitro* studies	NO	Low	Botthoulath et al., 2025
732	Korea	Fermented food		2024	No virulence factors, toxin-production-related genes, or biogenic amine synthesis genes detected.	*In vitro* studies	YES	Low	Choi et al., 2025
						*In vitro* studies	YES		
CECT8330 (KABP041)	China	Infant intestines	3.6.4 Clinical tolerability and oral safety	2021	No treatment-related side effects, adverse events, or unexpected effects were reported during the 21-days intervention.	Clinical study	YES	High	Chen et al., 2021
GH	Czech Republic	Human-stemmed lines		2023	Safety interpretability was limited by multiple confounding factors, precluding strain-specific attribution of the observed safety profile.	Clinical study	NO	High	Bartos et al., 2023
L28	Japan	Longan fruit		2016	Short-term oral tolerability reported in obese patients.	Clinical study	YES	High	Higashikawa et al., 2016
KID7	Korea	Fermented finger millet		2026	Adverse event rate of 16% in MASLD patients, including abdominal pain, distension, constipation, diarrhea, nausea, and urticaria; none progressed to serious adverse events.	Clinical study	YES	High	Won et al., 2025
IDS885	Japan	Fermented food		2018	No adverse reactions or serious adverse events attributable to the intervention were reported over 8 weeks in patients with mild-to-moderate active ulcerative colitis.	Clinical study	YES	High	Bamba et al., 2018

### 3.2 Antioxidant

Oxidative stress denotes an imbalance between the generation of reactive oxygen species (ROS) and the organism’s antioxidant capacity to eliminate ROS (Tumilaar et al., 2024). ROS can be categorized into two types: free radicals and non-free radicals. Examples of free radicals include superoxide radicals (O_2_∙-), hydroxyl radicals (OH), and 2,2-Diphenyl-1-picrylhydrazyl (DPPH), among others. Notably, DPPH is a stable synthetic free radical widely used *in vitro* to evaluate free radical scavenging activity. Non-radical forms encompass hydrogen peroxide (H_2_O_2_), singlet oxygen (^1^O_2_), and ozone (O_3_) (Łepecka and Kołożyn-Krajewska, 2025). Chronic oxidative stress may result in multisystem disorders including Alzheimer’s disease (Calabrese et al., 2018), Parkinson’s disease (Calabrese et al., 2018), cardiovascular disease (Rana and Singh, 2018), chronic obstructive pulmonary disease (Domej et al., 2014), chronic kidney disease (Zuo et al., 2019), and cancer (Davalli et al., 2018). *P. pentosaceus*, a member of lactic acid bacteria, possesses four primary antioxidant processes that are intricately linked.

#### 3.2.1 Direct clearance of reactive oxygen species (ROS)

The ROS-scavenging ability of *P. pentosaceus L1* may be ascribed to its cell wall components, including peptidoglycan, teichoic acid, lipoteichoic acid, and its extracellularly released EPS. This work demonstrated that the fermentation supernatant possessed markedly superior antioxidant activity compared to the cell-free extract, suggesting that extracellular metabolites are pivotal in free radical scavenging. Considering the strain’s substantial EPS-producing capacity and the corroborating genomic data, it is plausible to postulate that its ROS-scavenging activity is intricately linked to EPS secretion (Mustafa et al., 2025).

#### 3.2.2 Production of antioxidant enzymes

Research conducted by Zhang et al. (2021) showned that moderate oxidative stress generated by H_2_O_2_ elevated the intracellular activities of superoxide dismutase (SOD) and glutathione peroxidase (GSH-Px) in *P. pentosaceus R1*. This improvement enhanced the strain’s scavenging efficacy against DPPH, ∙OH, and O_2_∙– radicals (Zhang et al., 2021).

#### 3.2.3 Regulating oxidative processes in the body

*Pediococcus pentosaceus* SMM914 can activate the Nrf2-Keap1 antioxidant signaling pathway in hepatocytes. By inhibiting KEAP1, the strain promotes nuclear translocation of Nrf2. Once in the nucleus, Nrf2 binds to the antioxidant response element (ARE), which upregulates the expression of downstream antioxidant enzymes, including NQO-1, HO-1, SOD1, and Catalase (CAT). In parallel, the activities of GSH-Px, SOD, and CAT are increased, while the level of MDA, a marker of lipid peroxidation, is significantly reduced (Wang L. et al., 2022). *P. pentosaceus* PP04 activates the Nrf2 signaling pathway and downregulates CYP2E1, a metabolic enzyme responsible for ROS generation, thereby reducing ROS production (Wang Y. et al., 2021). *P. pentosaceus* MG9015 markedly suppressed the expression of inducible nitric oxide synthase (iNOS) and decreased nitric oxide (NO) production. The findings indicate that the strain may have anti-inflammatory capabilities; however, additional *in vivo* investigations are required to validate this effect (Kim K. T. et al., 2021).

#### 3.2.4 Chelation of transition metal ions

*Pediococcus pentosaceus* may alleviate oxidative stress by chelating transition metal ions, including iron and copper. This process is proposed to diminish hydroxyl radical (OH) production by inhibiting the Fenton reaction. Nevertheless, additional research is required to validate this effect (Łepecka et al., 2025).

#### 3.2.5 Summary

Overall, *P. pentosaceus* demonstrates significant antioxidant capacity and may assist in alleviating oxidative stress-related damage. Its antioxidant properties are evident not only through direct *in vitro* free radical scavenging but also through the modulation of host redox homeostasis and inflammatory responses. Nevertheless, existing evidence predominantly relies on *in vitro* investigations. A systematic evaluation criterion for strain-specific potency remains absent, and the underlying molecular mechanisms necessitate additional elucidation. Future study should aim to clarify the mechanisms and do clinical investigations to more accurately delineate its therapeutic potential in disorders associated with oxidative stress. The summary of strains and evidence grading for this part are presented in Table 1. The mechanistic diagram is shown in Figure 2B.

### 3.3 Secretion of bacteriocins and bacteriocin-like inhibitory substances

#### 3.3.1 Bacteriocins

Bacteriocins are antimicrobial proteins or peptides synthesized by bacteria that inhibit or eliminate other bacteria, especially those of the same or closely related species (Begum et al., 2019). Their antibacterial activities are mostly accomplished by enhancing cell membrane permeability or compromising membrane integrity, resulting in the death of target cells. Additionally, as bacteriocins are proteins, they are readily destroyed by proteases in the digestive system, hence diminishing the likelihood of resistance development (Anjana and Tiwari, 2022; Darbandi et al., 2022). Recent studies have shown that *P. pentosaceus* can secrete bacteriocins, which inhibit or kill various bacteria. The specific bacterial targets may be associated with the isolation site of the strain; however, this relationship has not yet been confirmed by extensive research. Studies have confirmed that *P. pentosaceus 732* exhibits anti-*Listeria* activity mediated by secreted bacteriocins; however, the specific bacteriocin components responsible have not yet been definitively identified (Choi et al., 2025). *P. pentosaceus E3*, isolated from shrimp gut, encodes the bacteriocins pediocin A and penocin A, which are likely the primary antimicrobial agents contributing to its inhibitory efficacy, especially against *Listeria monocytogenes* and *Staphylococcus aureus* (Zaghloul and Halfawy, 2024). *P. pentosaceus ST3633BG* inhibits various strains of *Listeria monocytogenes* and vancomycin-resistant *Enterococcus*. Its antibacterial activity is most likely associated with the secretion of pediocin PA-1, although this bacteriocin was not purified or independently characterized in the study. In addition, *ST3633BG* exhibits optimal bacteriocin secretion at 37 °C (Fugaban et al., 2022).

#### 3.3.2 Bacteriocin-like inhibitory substances

Bacteriocin-like inhibitory substances (BLIS), a category of antimicrobial peptides, can exert antibacterial activities via various methods. These substances suppress or eliminate microorganisms by obstructing DNA and RNA synthesis in target bacteria or compromising cell membrane integrity (van Zyl et al., 2020; da Silva et al., 2024). The *P. pentosaceus MT323062*, isolated from the vaginal discharge, showed bactericidal activity against *Enterococcus faecalis MW051601*, a common pathogen that causes aerobic vaginitis (AV). This activity was verified to be associated with BILS produced by the strain (Shazadi and Arshad, 2022). Furthermore, the BLIS produced by *P. pentosaceus LBM 18*, isolated from maize silage, exhibits inhibitory activity against *Lactobacillus sakei*, *Enterococcus faecium*, *Listeria innocua*, *Listeria seeligeri*, and multiple silage-associated fungi. This BLIS is considered a proteinaceous compound that may also contain a saccharide moiety contributing to its activity. Its antimicrobial effects are likely mediated by its hydrophilic and amphipathic properties, which facilitate interaction with target cell membranes and lead to membrane disruption (de Souza de Azevedo et al., 2020). *P. pentosaceus* Li05 (CGMCC 7049) may contribute to gut microbiota homeostasis by upregulating the antimicrobial peptide Reg3β and reducing the relative abundance of ethanol-associated bacterial genera, including *Staphylococcus*, *Oscillospira*, and *Allobaculum* (Jiang et al., 2020). Currently, research on the specific types and optimal dosages of bacteriocins remains limited. For example, *P. pentosaceus KABP041* has shown clinical potential in alleviating infantile colic (IC) when used in combination with *Bifidobacterium longum KABP042*. This effect is associated with the secretion of the antimicrobial peptide Bisin and an uncharacterized bacteriocin (Moreno-Villares et al., 2024).

#### 3.3.3 Summary

Overall, current evidence indicates that bacteriocin or BLIS production is a key antimicrobial property of *P. pentosaceus*, primarily through membrane disruption, thereby supporting its probiotic potential against specific pathogens. However, the precise active components, strain-specific variations, optimal dosage, and clinical relevance remain insufficiently characterized. Stronger mechanistic and translational studies are still needed. The summary of strains and evidence grading is presented in Table 1, and the proposed mechanism is illustrated in Figure 2C.

### 3.4 Anti-inflammatory and immunomodulatory effects

*Pediococcus pentosaceus* helps maintain host homeostasis by reducing lipopolysaccharide (LPS)-induced inflammatory responses, preserving intestinal barrier integrity, promoting short-chain fatty acid (SCFAs) production in the gut and modulating immune cell activity and cytokine secretion (Cristofori et al., 2021).

#### 3.4.1 Reduce lipopolysaccharide-induced inflammatory responses

Recent studies have shown that even trace amounts of lipopolysaccharide (LPS) from Gram-negative bacteria are sufficient to trigger inflammatory responses (Jiang et al., 2021). LPS predominantly derives from pathogenic gut bacteria, and when the integrity of the intestinal barrier is impaired, LPS readily infiltrates the bloodstream (Hutchinson et al., 2020; Wang G. et al., 2021). An *in vitro* study demonstrated that *P. pentosaceus* AK-23 harbors a cell wall-associated protein containing motifs resembling LPS-binding and hydrophobic-binding domains. This protein attenuates LPS toxicity by promoting the release of fatty acids–including hydroxymyristic acid, palmitic acid, and stearic acid–as well as glucose from LPS, an effect that may be associated with hydrolysis of the bonds linking fatty acids to sugar chains within the LPS molecule. LC-MS/MS-based homology analysis further revealed that this protein shares 56% sequence homology with the ATP-binding subunit of Clp protease; however, its precise enzymatic mechanism remains to be fully elucidated (Asami et al., 2017). Studies have shown that EPS on the surface of probiotics can inhibit LPS-induced inflammatory responses (Angelin and Kavitha, 2020). Although direct evidence on LPS inhibition by EPS from *P. pentosaceus* is currently lacking, *P. pentosaceus 4412*, isolated from fermented *Manilkara zapota* juice, produces cell-surface EPS that suppress pro-inflammatory cytokines (IL-1β, IL-6, and TNF-α) while promoting the anti-inflammatory cytokine IL-10, thereby exerting anti-inflammatory effects (Angelin and Kavitha, 2025).

#### 3.4.2 Preserve intestinal barrier integrity

The intestinal barrier function consists of mechanical barrier (tight junction proteins like ZO-1 and occludin), chemical barrier (mucus layer), microbial barrier (modulate beneficial gut microbiota), and immunological barrier (reduced TNF-α and IL-6). Once compromised, it can let intestinal endotoxins (such as LPS) to enter the bloodstream through the interstices, eliciting a systemic inflammatory response (Zhong et al., 2025). An *in vivo* zebrafish study showed that *P. pentosaceus* PR-1 improves intestinal barrier integrity by increasing the expression of occludin, claudin-1, and ZO-1, possibly in association with its regulatory effects on the gut microbiota (Liu et al., 2023). *P. pentosaceus* IM96 increases the number of goblet cells in the intestinal mucosa and enhances MUC-2 (gel-forming mucin/mucin-2) secretion, which may help restore mucosal barrier function. However, the specific signaling pathways involved in MUC-2 upregulation were not investigated in this study (Li et al., 2021). Another study on *P. pentosaceus* LI05 (CGMCC 7049) showed that, in a DSS-induced colitis model, LI05 upregulated the intestinal epithelial tight junction marker Tjp1 (the gene encoding ZO-1). This effect was accompanied by an increased abundance of beneficial bacteria, including Verrucomicrobiaceae, Verrucomicrobia, and *Akkermansia*. These changes suggest that LI05 may promote intestinal mucosal barrier repair by improving gut microecological homeostasis. In addition, LI05 reduced the levels of pro-inflammatory cytokines such as TNF-α and IL-6, thereby potentially attenuating inflammation-associated damage to epithelial tight junctions and improving intestinal barrier function (Bian et al., 2020).

#### 3.4.3 SCFAs-mediated barrier and immune regulation

Short-chain fatty acids, such as acetic acid, propionic acid, and butyric acid, have been thoroughly investigated and demonstrated to play a role in the regulation of immune cells and cytokines, as well as in preserving the integrity of intestinal barrier function (Golpour et al., 2023). An animal study of *P. pentosaceus* Li05 (CGMCC 7049) showed that this strain increased intestinal SCFAs levels. Given the positive correlation between SCFAs levels and ZO-1 expression, SCFAs may contribute to the restoration of intestinal barrier function (Jiang et al., 2020). Another review has noted that SCFAs, particularly butyric acid, serve as a major energy source for intestinal epithelial cells. This may represent one of the potential mechanisms by which SCFAs promote ZO-1 expression (Xiong et al., 2022). In a zebrafish model, chronic administration of *P. pentosaceus* YC elevated intestinal butyrate levels, likely through gut microbiota remodeling. This metabolic shift activated the NLRP3 inflammasome signaling pathway, promoting local neutrophil recruitment via the butyrate–NLRP3–IL-1β axis and ultimately enhancing the host’s anti-inflammatory capacity (Shan et al., 2021).

#### 3.4.4 Modulate immune cell activity and cytokine secretion

Other metabolites produced by *P. pentosaceus* may also contribute to anti-inflammatory regulation. *P. pentosaceus* restores intestinal indole metabolites, including indole-3-propionic acid (IPA), indole-3-acetic acid (IAA), and indole-3-aldehyde (IA), which are considered potential aryl hydrocarbon receptor (AhR) ligands. Activation of AhR helps alleviate systemic inflammatory responses. However, this mechanism has not yet been directly validated experimentally (Yu et al., 2021). *P. pentosaceus* may alleviate systemic inflammatory responses by modulating immune cells and cytokine responses. In immune cell-based studies, *P. pentosaceus* KF159 promotes the differentiation of tolerogenic dendritic cells (tol-DCs) by upregulating key immunomodulatory molecules, including ICOS-L, PD-L1, and IDO. These tol-DCs enhance IL-10 production either directly or by stimulating CD4^+^CD25^+^Foxp3^+^ regulatory T cells to secrete IL-10, thereby exerting potent anti-inflammatory effects (Eom et al., 2024).

The anti-inflammatory effects of *P. pentosaceus* are highly strain- and context-dependent. In metabolic and liver-related inflammatory models, *P. pentosaceus KCTC 18308P* and *PP04* suppressed MAPK/NF-κB- and TLR4/MyD88/NF-κB-associated signaling, respectively, reducing hepatic and systemic inflammatory responses (Wang Y. et al., 2021; Yu et al., 2021). In colitis models, KFT-18-derived EPS inhibited NF-κB- and STAT1-associated signaling and reduced IL-6 and IL-1β production (Lee J. H. et al., 2022). In a 5-FU-induced intestinal mucositis model, *P. pentosaceus PP34* downregulated NLRP3-, CXCL- 5-, and CXCL-9-associated inflammatory responses (He et al., 2025). In cigarette smoke- and ozone-induced COPD mouse models, *P. pentosaceus SMM914* activates the tryptophan–melatonin metabolic pathway, thereby enhancing endogenous antioxidant metabolism and alleviating pulmonary oxidative stress. Meanwhile, it suppresses Hspa1a-, Hspa1b-, TLR2-, and TLR4-associated signaling pathways, thereby reducing M1 macrophage polarization and inflammatory responses (Liu Y. et al., 2024). Collectively, these findings indicate that *P. pentosaceus* does not regulate inflammation through a single universal pathway; rather, its effects are dependent on the strain, disease model, and experimental context.

#### 3.4.5 Summary

In summary, *P. pentosaceus* regulates host inflammatory homeostasis through multiple interconnected mechanisms rather than a single pathway. It acts via a comprehensive network involving reduction of pro-inflammatory stimuli, reinforcement of the intestinal barrier, modulation of microbial metabolism, and reshaping of immune responses. These processes are closely interrelated and collectively maintain gut-immune axis homeostasis by limiting the systemic dissemination of intestinal-derived inflammatory signals, thereby contributing to the alleviation of systemic inflammation. However, current research remains predominantly based on animal experiments and mechanistic inferences; significant variations in effects exist among different strains, and direct validation is still lacking for certain key steps–particularly regarding the causal relationships between specific metabolic products or stress-related molecules and anti-inflammatory outcomes, which remain insufficiently elucidated. Consequently, further direct evidence is required to confirm these findings. The summary of strains and evidence grading is presented in Table 1, and the proposed mechanism is illustrated in Figure 2D.

### 3.5 Antibacterial, antifungal and antiviral

The ability of probiotics to counteract pathogens represents a key evaluation criterion, with growing evidence supporting their applications in cosmetic antibacterial agents (Oh et al., 2006), food preservatives (Cheruvari and Kammara, 2025), and adjunctive therapeutics (Fang et al., 2019).

#### 3.5.1 Antibacterial

The antibacterial properties of *P. pentosaceus* operate through both direct and indirect mechanisms. Direct effects include: (1) bacterial metabolites that eliminate or suppress the proliferation of pathogens; and (2) the obstruction of adhesion, colonization, and biofilm development of harmful bacteria via competitive inhibition (Sharma and Lee, 2025). Indirect effects, mediated through host–probiotic interactions, encompass: (1) augmenting epithelial barrier function; (2) enhanced binding to intestinal mucosa, which physically displaces harmful microbes; and (3) modulating the immune system (Bermudez-Brito et al., 2012). *P. pentosaceus* exhibits broad-spectrum antibacterial activity, with its inhibitory mechanisms varying depending on the target pathogen. *P. pentosaceus 2–5*, isolated from the chicken intestinal tract, inhibits the growth of *E. coli*, *S.* Typhimurium, and *S. aureus* via its cell-free supernatant (CFS). The effect may attributed to extracellular antimicrobial substances secreted into the CFS, particularly organic acids. The strain also inhibited *S*. Typhimurium adhesion to Caco-2 cells in competition and exclusion assays, an effect that may be partly attributable to its relatively strong epithelial adhesion capacity, which could help limit pathogen attachment to the intestinal surface (Wang P. et al., 2022). Bacterial quorum sensing (QS) is a density-dependent communication system that enables bacteria to sense population density and coordinate collective behavior. As autoinducers accumulate and reach a threshold concentration, they trigger downstream gene expression, promoting bacterial virulence and pathogenicity. The Las, Rhl, and PQS systems form major QS circuits in *P. aeruginosa* and regulate multiple virulence-associated traits. The HAQ/PQS system is a major quorum-sensing pathway in *P. aeruginosa* that regulates virulence-associated phenotypes, especially biofilm formation, while also controlling the production of several virulence factors, including pyocyanin, rhamnolipid, pyoverdine, and extracellular enzymes such as proteases and lipases (Miranda et al., 2022). Boujnane et al. (2025) showed that *P. pentosaceus MZF16* attenuated the quorum-sensing activity of *Pseudomonas aeruginosa H103* (*P. aeruginosa H103*) by significantly reducing the production of 2-alkyl-4-quinolones (HAQs), including PQS-associated signaling molecules. Disruption of this signaling pathway may reduce the virulence of *P. aeruginosa H103* without directly suppressing bacterial growth. Additionally, the authors identified pyoverdine–a key siderophore mediating host iron acquisition during infection–as an additional mechanism by which this strain may impair bacterial fitness and infectivity (Boujnane et al., 2025). Furthermore, immunomodulation can also confer indirect antibacterial effects. As previously described, *P. pentosaceus YC* enhances host resistance to *Aeromonas hydrophila* infection through the butyrate–NLRP3–IL-1β axis (Shan et al., 2021).

#### 3.5.2 Antifungal

The antifungal properties of *P. pentosaceus* are chiefly utilized to suppress foodborne fungus, aiding in the prolonged preservation of food (Fischer et al., 2025). *P. pentosaceus ST3633BG* exhibited inhibitory activity against several spoilage molds commonly associated with fruits, vegetables, and grains, including *Alternaria alternata*, *Aspergillus flavus*, and *Aspergillus niger*. This antifungal activity may be attributed to lactic acid and other organic metabolites secreted by the strain, including benzoic acid, 2-hydroxyisocaproic acid, β-phenyllactic acid, α-hydroxybutyric acid, and 1,3-butanediol (Fugaban et al., 2022). Additionally, *P. pentosaceus C15* inhibited the growth of several toxigenic fungi *in vitro*, particularly mycotoxin-producing molds associated with meat spoilage, such as *Aspergillus* and *Penicillium*. This antifungal activity is attributed to the production of organic acids (lactic and acetic acid), phenolic acids (vanillic and syringic acid), and volatile organic compounds (VOCs) such as phenylethyl alcohol, nonanoic acid, and octanol derivatives (Nazareth et al., 2023). However, whether these substances act individually or in combination has yet to be established. Consequently, *P. pentosaceus* demonstrates potential as a viable biopreservative to substitute or diminish the reliance on chemical preservatives. The antifungal efficacy of probiotics is concurrently influenced by environmental temperature. Mateo et al. (2023) reported that *P. pentosaceus M9MM5b, S11sMM1, and S1M4* exhibited temperature-dependent antifungal activity against toxigenic *Fusarium* spp. In dual-culture assays, all three strains demonstrated strong inhibition at 30 °C, with inhibitory rates ranging from 56.1% to 100% (Mateo et al., 2023). Muhialdin et al. (2011) reported that *P. pentosaceus Te010* produced heat-stable antifungal metabolites capable of delaying fungal spoilage in bread, suggesting potential applications as biopreservatives in bakery products.

#### 3.5.3 Antiviral

Three common antiviral mechanisms have been reported: (1) directly adsorb and capture viruses; (2) contact with the immune system; and (3) synthesis of antiviral agents (Farahmandi et al., 2023). Heat-inactivated *P. pentosaceus VM95* and VM21, isolated from human milk, inhibited R5-tropic *HIV-1* infection *in vitro* at rates of 49.0% and 45.5%, respectively. Since this activity was associated with intact bacterial cells rather than soluble metabolites, the authors hypothesized that surface-associated peptidoglycans and EPS may mediate this effect by capturing *HIV-1* and preventing target cell contact. Nevertheless, this proposed mechanism awaits experimental verification (Martín et al., 2010). Using a mouse influenza model, Chen et al. (2024) demonstrated that oral administration of *P. pentosaceus MIANGUAN2* reduced pulmonary viral load and attenuated lung injury. These effects appear to be mediated through immunomodulatory and gut microbiota-dependent mechanisms, particularly via enhanced intestinal SCFAs production and modulation of the gut–lung axis. Rather than directly neutralizing the virus, SCFAs likely support host antiviral defense by suppressing excessive pulmonary inflammation through downregulation of TNF, apoptosis, and NF-κB signaling pathways, accompanied by reduced pro-inflammatory cytokines, including TNF-α, IL-1β, IFN-γ, and IL-12p70. Notably, SCFAs levels were negatively correlated with both pulmonary viral load and pro-inflammatory cytokine levels, further supporting this proposed mechanism (Chen et al., 2024). Studies suggest that probiotics may exert antiviral effects through lactic acid secretion, primarily by creating an acidic microenvironment that denatures viral surface proteins and/or disrupts the lipid envelope, thereby impairing viral adsorption and cell entry. These effects are therefore likely to predominate during the early stages of infection. Given that *P. pentosaceus* is known to produce lactic acid, this mechanism may also contribute to its antiviral activity. However, direct experimental validation in the context of *P. pentosaceus* remains lacking, representing a promising avenue for future research (Fugaban et al., 2022; Vilhelmova-Ilieva et al., 2022, 2023).

#### 3.5.4 Summary

Overall, the antibacterial, antifungal, and antiviral properties of *P. pentosaceus* are strongly strain-specific, generally involving antimicrobial metabolite production, interference with pathogen adhesion or virulence, and modulation of host barrier or immune responses. However, current evidence remains largely limited to *in vitro* studies and animal models, with key aspects–including active components, dose-response relationships, environmental stability, and metabolite interactions–yet to be fully characterized. Rigorous clinical and translational research is therefore needed to determine whether *P. pentosaceus* can be reliably developed as a biopreservative, adjunctive antimicrobial agent, or probiotic intervention for pathogen-related diseases. The summary of strains and evidence grading is presented in Table 1, and the proposed mechanism is illustrated in Figure 2E.

### 3.6 The safety of *Pediococcus pentosaceus*

Safety assessment is a critical component of probiotic screening, encompassing evaluation of hemolytic activity, antibiotic resistance, toxin production potential, the presence of antibiotic-resistance and pathogenic genes, clinical tolerability, and oral safety (Lee et al., 2024). Although *P. pentosaceus* strains are generally classified as low-risk under the EFSA’s Qualified Presumption of Safety (QPS) framework, given the strong strain specificity demonstrated across the studies reviewed above, independent safety validation remains necessary for each individual strain (Efsa Panel on Biological Hazards (BIOHAZ), Allende et al., 2026).

#### 3.6.1 Hemolytic activity

Hemolytic activity, defined as the capacity of bacteria to lyse erythrocytes and release hemoglobin, is a key safety indicator in probiotic evaluation. As explicitly stated in the 2002 FAO/WHO guidelines, three hemolytic patterns are recognized: α-hemolysis, involving partial hemoglobin reduction; β-hemolysis, characterized by complete erythrocyte lysis; and γ-hemolysis, indicating no hemolytic reaction. Absence of hemolytic activity has been reported across multiple *P. pentosaceus* strains, including BalaMMB-P3 (Park et al., 2022), CACC616 (Park et al., 2023), L1 (Mustafa et al., 2025), and 732 (Choi et al., 2025).

#### 3.6.2 Antibiotic resistance and the presence of antibiotic-resistance genes

Probiotics harboring transferable antibiotic resistance genes may transfer these genes to potentially pathogenic intestinal bacteria via mobile genetic elements–such as plasmids, transposons, integrons, or bacteriophages–upon entering the gut, thereby compromising the efficacy of clinical antibiotic treatments (von Wintersdorff et al., 2016). Chromosomally encoded resistance traits are generally considered non-transferable. In *P. pentosaceus* OBK05, resistance to kanamycin, streptomycin, vancomycin, ciprofloxacin, and norfloxacin was attributed to this non-transferable pattern, whereas trimethoprim resistance was plasmid-encoded and therefore potentially transferable (Bhukya and Bhukya, 2021). Another study shows, although *P. pentosaceus* E3 exhibited phenotypic resistance to several antibiotics–including teicoplanin, penicillin G, cefoxitin, sulfamethoxazole/trimethoprim, and colistin sulfate–genomic analysis revealed no clinically relevant antibiotic resistance genes or plasmid replication elements (Zaghloul and Halfawy, 2024). A multi-strain study similarly reported a discrepancy between phenotypic and genomic resistance profiles in *P. pentosaceus*. Many strains exhibited minimum inhibitory concentration (MIC) values–defined as the lowest antibiotic concentration required to visibly inhibit bacterial growth–for tetracycline and chloramphenicol that exceeded recommended microbiological cutoffs. However, whole-genome screening revealed no known antibiotic resistance determinants in any of the 35 strains tested (Shani et al., 2021). This highlights the importance of integrating phenotypic susceptibility testing with genomic screening, as phenotypic resistance alone may not accurately reflect the risk of transferable antibiotic resistance.

#### 3.6.3 Toxin production potential and the presence of pathogenic genes

Biogenic amines, produced primarily through microbial decarboxylation of amino acids, are the most frequently scrutinized toxic metabolites in the safety assessment of lactic acid bacteria and fermented foods. Excessive accumulation of biogenic amines within food products or the intestinal environment may trigger adverse reactions, including headaches, hypertension, and gastrointestinal discomfort. Relevant review articles indicate that the biogenic amines most commonly identified in fermented foods include histamine, tyramine, putrescine, and cadaverine, with histamine and tyramine regarded as posing the greatest safety concerns (Barbieri et al., 2019; Turna et al., 2024). Møller et al. (2020) reported that several cheese-derived *P. pentosaceus* isolates were capable of producing histamine *in vitro*, suggesting that this species may contribute to biogenic amine accumulation in aged cheese. This finding highlights a potential safety concern associated with certain *P. pentosaceus* strains in fermented dairy products (Møller et al., 2020). Hassan et al. (2024) demonstrated that inoculation with *P. pentosaceus K-B21* or *K-B01* during kisra (sourdough pancakes) fermentation completely inhibited tyramine and cadaverine formation, while markedly reducing levels of other biogenic amines, including putrescine, histamine, spermine, and spermidine (Hassan et al., 2024). Furthermore, virulence factors, toxin-production-related genes, and biogenic amine synthesis genes were not detected in certain strains, including *P. pentosaceus 732* and *BBS1* (Botthoulath et al., 2025; Choi et al., 2025). *P. pentosaceus BBS1* also harbors genes encoding β-glucosidase (linamarase; EC 3.2.1.21) and 6-phospho-β-glucosidase (EC 3.2.1.86), enabling hydrolysis of cyanogenic glycosides and cyanide detoxification during fermentation, consistent with demonstrated linamarase activity and cyanide tolerance (Botthoulath et al., 2025).

#### 3.6.4 Clinical tolerability and oral safety

Probiotics may be associated with adverse reactions, including systemic infections, gastrointestinal side effects, dermatological complications, endocarditis, gene transfer, deleterious metabolic effects, and immune dysregulation. Clinical tolerability and oral safety warrants particular attention in vulnerable populations, including infants, the elderly, and immunocompromized individuals (Sotoudegan et al., 2019). In this randomized, double-blind, placebo-controlled trial, no treatment-related side effects, adverse events, or unexpected effects were reported over the 21-days intervention with a combined probiotic preparation containing *Bifidobacterium longum* CECT7894 (KABP042) and *P. pentosaceus CECT8330 (KABP041)*. However, as the two strains were administered in combination, the safety profile of each individual strain could not be independently assessed (Chen et al., 2021). In another multi-strain formulation study evaluating *P. pentosaceus GH* for memory and cognitive improvement, the interpretability of safety findings was limited by multiple confounding factors, making it difficult to attribute the observed safety profile specifically to *P. pentosaceus GH* (Bartos et al., 2023). Single-strain studies provide more direct, albeit still limited, evidence. Short-term oral tolerability of *P. pentosaceus L28* was reported in obese patients (Higashikawa et al., 2016). In patients with MASLD, administration of *P. pentosaceus KID7* was associated with an adverse event rate of 16%, primarily comprising abdominal pain, abdominal distension, constipation, diarrhea, nausea, and urticaria; none of these events progressed to serious adverse events (Won et al., 2025). In patients with mild-to-moderate active ulcerative colitis, an 8-weeks intervention with *P. pentosaceus IDS885* did not result in any adverse reactions or serious adverse events clearly attributable to the intervention (Bamba et al., 2018).

#### 3.6.5 Summary

Overall, current evidence suggests that *P. pentosaceus* has a generally favorable safety profile, particularly with respect to the absence of hemolytic activity across many evaluated strains and the limited detection of clinically relevant virulence factors or transferable antibiotic resistance genes. However, safety cannot be extrapolated at the species level given the marked strain specificity of *P. pentosaceus*, necessitating an integrated evaluation of phenotypic traits, genomic risk factors, toxic metabolite production, and clinical tolerability for each individual strain. The primary limitations of current evidence include the predominance of *in vitro* and genomic data, a limited number of clinical studies, short intervention durations, small sample sizes, and frequent use of multi-strain formulations that preclude attribution of safety outcomes to *P. pentosaceus* alone. Adverse event reporting also remains inconsistent across clinical studies, and evidence in vulnerable populations is insufficient. Future research should therefore incorporate standardized safety endpoints, extended follow-up periods, whole-genome screening, and well-designed single-strain clinical trials to better characterize the strain-specific oral safety and translational potential of *P. pentosaceus*. The summary of strains and evidence grading is presented in Table 1, and the proposed mechanism is illustrated in Figure 2F.

## 4 Therapeutic potential of *Pediococcus pentosaceus* in systemic diseases

Building on the mechanisms outlined above, this section examines the potential clinical translation of *P. pentosaceus* across various disease contexts. The summary of strains and evidence grading for this part are presented in Table 2. The mechanistic diagram is shown in Figure 1.

**TABLE 2:** Therapeutic potential of *Pediococcus pentosaceus* in systemic diseases.

Strain	Nation	Source	Diseases	Year	Mechanism	Study type	Direct experimental validation	Evidence-support classification	References
KCTC 18308P	Korea	Finger millet (*Eleusine coracana*) gruel	4.1.1 MASLD	2021	Normalizes the F/B ratio → restores intestinal metabolic profiles (SCFAs, indole metabolites, bile acids) → maintains intestinal mechanical barrier integrity + suppresses hepatic macrophage activation and MAPK/NF-κB signaling.	In animal studies	NO	Moderate	Yu et al., 2021
PP04	China	Fermented food		2021	Restores intestinal mechanical and mucus barriers → limits LPS translocation into circulation → suppresses TLR4–MyD88–NF-κB inflammatory signaling.	In animal studies	NO	Moderate	Wang Y. et al., 2021
			4.2.1 Obesity		Activates AMPK → inhibitory phosphorylation of ACC1 and SREBP-1 → downregulates FAS and SCD1 → suppresses *de novo* lipid synthesis.				
			4.2.2 Hyperchole sterolaemia	2025	Hydrolyses conjugated bile salts → disrupts enterohepatic circulation → stimulates hepatic bile acid resynthesis → increases cholesterol consumption.	In animal studies + *in vitro* studies	NO	Moderate	Wang et al., 2025
					Inhibits intestinal FXR/FGF15 pathway → reduces bile acid reabsorption.		NO		
					Downregulates hepatic HMGCR and SREBP-2 mRNA expression → suppresses cholesterol synthesis.		YES	High	
			4.4.3 Inflammation-induced insulin resistance		Ameliorate high-fat diet-induced insulin resistance.	In animal studies	YES		
LP28	Japan	Longan fruit	4.2.1 Obesity	2012	Downregulates CD36, SCD1, and PPARγ.	In animal studies	NO	Moderate	Zhao et al., 2012
		Longan fruit	4.2.1 Obesity	2016	Non-viable components or heat-stable bioactive substances (e.g., EPS) → anti-obesity effects.	Clinical study	NO	High	Higashikawa et al., 2016
K28	Korea	Hulled barley		2023	Downregulates PPARγ and C/EBPα; reduces CD36 and LPL; suppresses FAS and ACC.	*In vitro* studies	NO	Low	Seo et al., 2023
RBHZ3	China	/		2025	Conversion of GSLs to ITCs activates PPAR signaling.	In animal studies	NO	Moderate	Jiang L. et al., 2025
E24-168	Bangladesh	LAB	4.2.2 Hyperchole sterolaemia	2026	Downregulates FXR-related pathways and NPC1L1. Stimulates intestinal SCFAs production	In animal studies	NO	Moderate	Habib et al., 2026
			4.4.3 Inflammation-induced insulin resistance		Obesity → generates inflammatory factors + ROS → interferes with insulin signaling → insulin resistance.				
			4.6.2 Anxiety		Downregulates IL-1β, NLRP3, and Caspase-1 → restores HPA axis homeostasis → alleviates anxiety-like behavior.				
OBK05	India	Buttermilk	4.2.2 Hyperchole sterolaemia	2021	BSH activity → hydrolyses conjugated bile salts → disrupts enterohepatic circulation and BAs reabsorption → stimulates hepatic BAs resynthesis → increases cholesterol consumption.	*In vitro* studies	NO	Low	Bhukya and Bhukya, 2021
MT323062	Pakistan	Vaginal discharge	4.1.2 Other hepatobiliary diseases- liver disease	2025	Activate SOD and CAT.	In animal studies	YES	High	Hameed et al., 2025
			4.7 Other disease-cadmium poisoning		Promote intestinal excretion of cadmium.				
Li05 (CGMCC 7049)	China	Human intestines	4.1.2 Other hepatobiliary diseases-ALD	2020	Increases intestinal SCFAs (propionate and butyrate) + upregulates Reg3β→ restores intestinal mechanical barrier + limits harmful bacterial overgrowth → reduces gut-derived endotoxin exposure and bacterial translocation → attenuates hepatic TLR4-associated inflammatory responses.	In animal studies	NO	Moderate	Jiang et al., 2020
			4.1.2 Other hepatobiliary diseases- cholestasis	2023	Upregulates hepatic FXR–SHP + ileal FXR–FGF15 axes → inhibits Cyp7a1 → reduces hepatic bile acid synthesis.		NO	Moderate	Han et al., 2023
					Increases *Eubacterium* abundance → primary-to-secondary bile acid conversion.				
					Upregulates hepatic bile acid transporters + downregulates ileal reabsorption transporters → promotes fecal bile acid excretion.		YES	High	
			4.3.2 Functional gastrointestinal disorders-IBS	2024	Downregulates 5-HT3BR mRNA → reduces 5-HT3 receptor-mediated intestinal motility and secretion		NO	Moderate	Wu et al., 2024
				2025	Elevated BSH activity → hydrolyses conjugated bile acids into free bile acids → activates TGR5 receptors on enterochromaffin cells → upregulates TPH1 → promotes 5-HT biosynthesis and secretion.		NO	Moderate	Chen et al., 2025
Li05 (CGMCC 7049)	China	Human intestines	4.3.2 Functional gastrointestinal disorders-IBS	2025	Upregulates colonic MUC2 expression → enhances mucus secretion → lubricates intestinal lumen → facilitates fecal excretion.	In animal studies	YES	High	
B49	China	Human colostrum		2020	Increases intestinal SCFAs → elevates luminal osmotic pressure + improves fecal water content + activates 5-HT-related pathways	In animal studies	NO	Moderate	Huang et al., 2020
KABP041	Spain/Italy	Human intestines	4.3.2 Functional gastrointestinal disorders-infant colic	2021	Secretes lactic acid + antimicrobial peptides → inhibits unfavorable intestinal bacteria.	Clinical studies	NO	High	Astó et al., 2021
	Spain/Mexico			2024	Enhances intestinal barrier integrity → attenuates inflammatory responses.			High	Moreno-Villares et al., 2024
KFT-18	Korea	Kimchi	4.3.1 Ulcerative colitis (UC)	2022	EPS → downregulates IL-6 and IL-1β	In animal studies	YES	High	Lee J. H. et al., 2022
					EPS →inhibits NF-κB- and STAT1-associated signaling.				
M6	China	Horse intestines	4.3.1 Ulcerative colitis (UC)	2025	Reduces IL-1β and IL-6 → attenuates inflammatory responses.	In animal studies	YES	High	Cao et al., 2025
					Restores intestinal mechanical barrier → limits mucosal exposure to luminal antigens and bacterial toxins → reduces mucosal injury.		NO	Moderate	
CECT 8330	China	Human intestines		2022	Increases CD4^+^CD25^+^Foxp3^+^ Tregs in colonic lamina propria → promotes IL-10 production → maintains intestinal mucosal immune homeostasis.	In animal studies	NO	Moderate	Dong et al., 2022
					Restores mechanical barrier integrity + increases SCFAs + modulates inflammatory cytokine profiles.		YES	High	
				2023	Promotes M1→M2 macrophage polarization → reduces IL-1β		YES	High	Hua et al., 2023
					Inhibits NF-κB- associated signaling.		NO	Moderate	
					Suppresses ROS generation in intestinal epithelial cells				
SL4	Korea	Kimchi	4.3.3 Colorectal cancer	2021	p8 gene introduced into *P. pentosaceus* SL4 → live biotherapeutic delivery vehicle → protected GI transit → targeted intestinal release → sustained local p8 secretion.	In animal studies	NO	Moderate	Chung et al., 2021
E3	Egypt	Shrimp intestines		2025	EPS production → induces tumor cell apoptosis + disrupts TNF-related tumor growth signaling.	*In vitro* studies	NO	Low	Halfawy and Zaghloul, 2025
QJ-12	China	Human intestines		2025	Secretes EPS → targets SAC gene → arrests tumor cells at mitotic metaphase.	*In vitro* studies	YES	Low	Liu et al., 2025
GS4	India	Khadi	4.3.3 Colorectal cancer	2016	Biohydrogenates LA → produces CLA → downregulates NF-κB and PI3K–Akt pro-survival signaling.	*In vitro* studies	NO	Low	Dubey et al., 2016
IM96	China	Turnip rape	4.3.4 Infectious diarrhea	2021	Directly inhibits EHEC O157:H7 growth.	*In vitro* studies	YES	Low	Li et al., 2021
					Increases intestinal SCFAs + restores mechanical and mucosal barriers → alleviates EHEC-induced intestinal inflammation.	In animal studies	NO	Moderate	
SNF15	China	Calf intestines		2025	Produces BLIS + hydrogen peroxide → directly suppresses *E. coli* K99	*In vitro* studies	YES	Low	Su et al., 2025
					Downregulates jejunal IL-6, IL-1β, and TNF-α (mRNA and protein)	In animal studies	YES	High	
					Restores intestinal mucus and mechanical barriers		NO	Moderate	
SMFM2016-WK1	Korea	Kimchi		2025	Secretes BLIS + organic acids → directly inhibits *E. coli*	*In vitro* studies	YES	Low	Cho et al., 2025
					Restores intestinal mechanical barrier → protects barrier integrity → attenuates host inflammatory responses.	In animal studies	NO	Moderate	
GT001	China	/		2024	Reduces intestinal *Salmonella* load	In animal studies	YES	High	Bumbie et al., 2024
					Increases serum IgA, IgG, and IgM		YES		
					Strengthens antioxidant defenses		NO	Moderate	
KI62	Korea	Kimchi	4.4.1 α-Amylase and α-glucosidase	2021	Inhibit α-amylase and α-glucosidase.	*In vitro* studies	YES	Low	Kim S. et al., 2021
MF000957	UAE	Camel milk	4.4.2 GLP-1	2024	Ferments milk proteins → produces bioactive peptides → inhibits DPP-IV → reduces GLP-1 degradation → prolongs GLP-1 activity.	*In vitro* studies	YES	Low	Mudgil et al., 2024
MIANGUAN2	China	Human intestines	4.5 Respiratory system diseases	2024	Enrichment of SCFA-producing Lachnospiraceae → potential enhancement of SCFA-mediated immunomodulation → suppression of TNF/NF-κB-related inflammatory signaling.	In animal studies	NO	Moderate	Chen et al., 2024
SMM914	China	Sow milk		2024	Enrichment of SCFA-producing Lachnospiraceae → potential enhancement of SCFA-mediated immunomodulation →suppression of Hspa1a/TLR4- related inflammatory signaling.	In animal studies	NO	Moderate	Liu Y. et al., 2024
ON81A	Korea	Fermented food		2021	Fermenting germinated *Rhynchosia nulubilis* by *Pediococcus pentosaceus* ON81A → production of GR-ON81A enriched in polyphenolic antioxidants → enhanced DPPH free radical.	*In vitro* studies	YES	Low	Lee and Park, 2021
WMU002	China	/	4.6.1 Parkinson’s disease	2022	Modulates Keap1/Nrf2 antioxidant signaling pathway → attenuates neuronal oxidative damage.	In animal studies	NO	Moderate	Pan et al., 2022
					Increases brain GABA levels (precise mechanism unclear).		YES	High	
ENM104	Thailand	Fermented food		2024	Lacks *gadA*/*gadB* genes + carries only *gadC* (glutamate/GABA antiporter) → limited GABA-producing capacity.	*In vitro* studies	YES	Low	Kompramool et al., 2024
WB3812	Korea	Fermented food	4.6.3 Alzheimer’s disease	2025	Nrf2-mediated antioxidant defenses ↑→ ROS accumulation ↓→ Bax/Bcl-2 ratio ↓ + mitochondrial apoptotic pathway stabilized + caspase-9/caspase-3 cascade inhibited → H_2_O_2_-induced neuronal apoptosis ↓	*In vitro* studies	NO	Low	Lee et al., 2025
YF01	China	Fermented food	4.7 Other disease- exercise capacity	2024	Upregulates MyHC I, SIRT1, and PGC-1α mRNA in muscle tissue → improves intramuscular energy metabolism.	In animal studies	NO	Moderate	Yang et al., 2024
					Upregulates SOD1, SOD2, and CAT in skeletal muscle and liver → enhances antioxidant capacity → alleviates exercise-induced oxidative stress.				
AF2	Türkiye	*Herniaria glabra* L.	4.7 Other disease- wound repair	2024	Live cells + lysates → promotes fibroblast migration + releases bioactive metabolites → accelerates scratch closure.	*In vitro* studies	NO	Low	Ugras et al., 2024
ZJUAF-4	China	Pickled vegetables	4.7 Other disease- paraquat poisoning	2021	Restores jejunal villus and microvillus integrity.	In animal studies	YES	High	Hao et al., 2021
					Enhances SOD-mediated antioxidant defense.				
					Activates Nrf2- related signaling pathway.		NO	Moderate	
732	Korea	Fermented food	4.7 Other disease- oral health	2025	Good survival under simulated oral conditions.	*In vitro* studies	YES	Low	Choi et al., 2025
					Resistance to most commercially available medications and oral care products.				
PP06	China	LAB	4.7 Other disease- breast cancer	2024	Combined with DOX → upregulates TNF-α, IFN-γ, and IFN-β→ activates CD8^+^ T cells + enhances macrophage function → enhances local anti-tumor immune responses.	In animal studies	YES	High	Ye et al., 2024
AOA2017	Korea	Finger millet	4.7 Other disease- atherosclerosis	2019	Fermentation → increases bioactive phenolic acids in p-coumaric acid→ reduces LPS-induced intracellular ROS accumulation in HUVECs → downregulates VCAM-1 expression → reduces THP-1 monocyte adhesion to endothelial cells.	*In vitro* studies	NO	Low	Kim et al., 2019
PP34	China	Bos grunniens intestines	4.7 Other disease- intestinal mucositis	2024	Increases T-SOD and GSH-Px levels → enhances systemic antioxidant capacity.	In animal studies	NO	Moderate	He et al., 2025
					Downregulates CXCL-9, CXCL-5, and NLRP3 expression → attenuates jejunal inflammation				

### 4.1 Hepatobiliary diseases

The gut-liver axis denotes the reciprocal communication mechanism between gut microbiota and its metabolites and the liver, facilitated by the portal vein, bile circulation, and systemic inflammatory and metabolic signals. Recent evidence demonstrates its significant involvement in safeguarding the liver and gallbladder (Tripathi et al., 2018; Pabst et al., 2023). In the gut–liver axis, injury is primarily driven by intestinal barrier dysfunction, increased translocation of gut-derived microbial products, and subsequent activation of hepatic inflammatory signaling. Increased intestinal permeability permits lipopolysaccharide (LPS) and other pathogen-associated molecular patterns (PAMPs) to enter the portal circulation, where they interact with hepatic pattern-recognition receptors–particularly Toll-like receptors (TLRs)–activating downstream inflammatory cascades, exacerbating hepatic oxidative stress, and ultimately contributing to hepatocyte injury (Manothiya et al., 2025). *P. pentosaceus* primarily exerts its effects within the gastrointestinal tract and may indirectly protect the liver and gallbladder through the gut–liver axis (Sanders et al., 2019).

#### 4.1.1 MASLD

Metabolic dysfunction–associated steatotic liver disease (MASLD), a prevalent liver condition, was rebranded from NAFLD to MAFLD in 2019 and subsequently to MASLD in 2023. Given that MASLD is diagnosed at the pathological level and encompasses a wider diagnostic range, the research findings on NAFLD and MAFLD may be deemed relevant to MASLD (Rinella and Sookoian, 2024; Song et al., 2024). *P. pentosaceus KCTC* 18308P normalizes the gastrointestinal Firmicutes-to-Bacteroidetes (F/B) ratio, thereby helping to restore intestinal metabolic profiles, including SCFAs, indole metabolites, and bile acids (BAs). This functional restoration is associated with maintained intestinal mechanical barrier integrity and suppression of hepatic macrophage activation and MAPK/NF-κB signaling, ultimately alleviating hepatic steatosis and inflammation (Yu et al., 2021). Although SCFAs are generally considered beneficial in MASLD, excessive SCFA production may promote hepatic free fatty acid (FFA) accumulation by inhibiting adenosine monophosphate-activated protein kinase (AMPK) activity (Hu et al., 2020). *P. pentosaceus PP04* restores the intestinal mechanical and mucus barriers, limiting LPS translocation from the gut into the circulation and suppressing subsequent activation of the TLR4/MyD88/NF-κB inflammatory signaling pathway, thereby indirectly alleviating high-fat diet-induced liver injury (Wang Y. et al., 2021).

#### 4.1.2 Other hepatobiliary diseases

*Pediococcus pentosaceus* Li05 (CGMCC 7049) may help restore the intestinal mechanical barrier by increasing intestinal SCFAs levels, particularly propionate and butyrate, while upregulating Reg3β expression to limit harmful bacterial overgrowth. These effects may reduce gut-derived endotoxin exposure and bacterial translocation, thereby attenuating hepatic TLR4-associated inflammatory responses and alleviating alcoholic liver disease (ALD) (Jiang et al., 2020). Moreover, the antioxidant pathways confer a protective impact on the liver. *P. pentosaceus MT323062* enhances antioxidant defenses by increasing SOD and CAT activities, thereby exerting hepatoprotective effects (Hameed et al., 2025). *P. pentosaceus Li05 (CGMCC 7049)* alleviates cholestasis through remodeling of BAs homeostasis via three mechanisms. First, it upregulates the hepatic FXR–SHP and ileal FXR–FGF15 signaling axes, inhibiting Cyp7a1 expression and reducing excessive hepatic BAs synthesis. Second, it modulates BAs transport by upregulating hepatic transporters (BSEP, MDR1, MDR2, and MRP2) while downregulating ileal reabsorption transporters (ASBT, OSTα/β, and MRP2), promoting fecal BAs excretion. Third, it increases the abundance of *Eubacterium*–which possesses bile salt hydrolase and 7α-dehydroxylation activity–facilitating conversion of primary to secondary BAs. Collectively, these mechanisms attenuate DDC-induced cholestatic liver injury (Han et al., 2023).

#### 4.1.3 Summary

Taken together, *P. pentosaceus* has demonstrated potential hepatobiliary protective effects in MASLD, ALD and cholestasis by targeting key pathological events along the gut–liver axis–including intestinal barrier disruption, microbial product translocation, hepatic inflammatory activation, oxidative stress, and BAs dysregulation. However, current evidence remains predominantly derived from animal and *in vitro* studies, and further clinical investigation is warranted.

### 4.2 Obesity and hypercholesterolaemia

The World Health Organization defines adult obesity as a body mass index (BMI) ≥ 30 kg/m^2^ and overweight as a BMI ≥ 25 kg/m^2^. Dyslipidaemia is characterized by abnormal plasma lipid or lipoprotein profiles, including elevated total cholesterol (TC), low-density lipoprotein cholesterol (LDL-C), triglycerides (TG), non-high-density lipoprotein cholesterol (non-HDL-C), or lipoprotein(a) [Lp(a)], alongside reduced high-density lipoprotein cholesterol (HDL-C). Hyperlipidaemia refers specifically to elevated circulating lipid levels. Obesity, particularly visceral obesity, promotes dyslipidaemia through adipose tissue insulin resistance, increased free fatty acid flux, enhanced hepatic very-low-density lipoprotein (VLDL) production, and impaired triglyceride clearance, commonly resulting in elevated TG, VLDL, apolipoprotein B (apoB), and non-HDL-C, with concurrent reductions in HDL-C. Notably, approximately 60%–70% of individuals with obesity have dyslipidaemia (Feingold, 2025, 2026).

#### 4.2.1 Obesity

*Pediococcus pentosaceus PP04* may reduce high-fat diet-induced lipid accumulation by activating AMPK-mediated lipid metabolic pathways. This activation promotes inhibitory phosphorylation of ACC1 and SREBP-1, downregulates lipogenesis-related genes including FAS and SCD1, and ultimately suppresses *de novo* lipid synthesis (Wang Y. et al., 2021). Via fermentation-mediated indirect effects, *P. pentosaceus RBHZ36* promotes the conversion of glucosinolates (GSLs) in broccoli stalk by-products into bioactive isothiocyanates (ITCs); PICRUSt functional prediction suggests these ITCs may attenuate adipose accumulation through PPAR-related signaling pathways (Jiang L. et al., 2025). In additional, the cellular extract of *P. pentosaceus K28* downregulates the core adipogenic transcription factors PPARγ and C/EBPα, reduces CD36 and LPL expression (fatty acid uptake), and suppresses FAS and ACC expression (fatty acid synthesis) (Seo et al., 2023). *P. pentosaceus LP28* downregulates hepatic CD36 (fatty acid uptake), SCD1 (fatty acid conversion and synthesis), and PPARγ (adipogenesis) (Zhao et al., 2012). Further RCTs demonstrated that heat-inactivated *P. pentosaceus* LP28 significantly reduced BMI, body fat percentage, body fat mass, and waist circumference in overweight patients, whereas live LP28 showed only a trend toward reduced body fat. This suggests that the anti-obesity effects of LP28 may not depend on colonization by viable bacteria, but rather on non-viable components or heat-stable bioactive substances, such as exopolysaccharides (Higashikawa et al., 2016).

#### 4.2.2 Hypercholesterolaemia

The strain *P. pentosaceus E24-168* may promotes cholesterol conversion by downregulating FXR-related pathways, thereby inhibiting cholesterol-to-BAs conversion. It also reduces dietary cholesterol absorption by downregulating NPC1L1, a key intestinal epithelial cholesterol uptake transporter. Additionally, the strain stimulates intestinal SCFA production, reduces hepatic lipid accumulation, and improves lipid metabolism in adipose tissue, although the specific underlying pathways remain to be elucidated (Habib et al., 2026). *P. pentosaceus* OBK05 has been shown to possess bile salt hydrolase (BSH) activity *in vitro*. By hydrolysing conjugated bile salts, BSH disrupts the enterohepatic circulation and BAs reabsorption, stimulating hepatic BAs resynthesis and consequently increasing cholesterol consumption, thereby exerting a potential cholesterol-lowering effect (Bhukya and Bhukya, 2021). *P. pentosaceus* PP04 possesses both BSH activity and the ability to inhibit intestinal FXR-related signaling, such as the FXR/FGF15 pathway. It also reduces hepatic expression of HMGCR–a key rate-limiting enzyme in cholesterol synthesis–and SREBP-2 mRNA, which regulates cholesterol synthesis. Through these mechanisms, PP04 effectively lowers blood cholesterol levels (Wang et al., 2025).

#### 4.2.3 Summary

In summary, current evidence suggests that *P. pentosaceus* holds potential therapeutic benefits for obesity and hypercholesterolaemia, primarily through regulation of lipid synthesis, fatty acid uptake, bile acid metabolism, and cholesterol absorption, as well as promotion of SCFAs production. However, findings remain largely strain-specific and are predominantly derived from animal studies, *in vitro* assays, functional predictions, or short-term clinical observations. Further experimental validation is therefore required to confirm the proposed mechanisms and potential clinical applications of specific strains.

### 4.3 Gastrointestinal diseases

Probiotics have shown therapeutic potential across a range of gastrointestinal conditions, including ulcerative colitis, functional gastrointestinal disorders, colorectal cancer, and infectious diarrhea, with outcomes closely linked to the regulatory mechanisms described in the preceding section (Wilkins and Sequoia, 2017; Latif et al., 2023).

#### 4.3.1 Ulcerative colitis (UC)

Ulcerative colitis (UC) is a chronic, persistent, or recurrent inflammatory bowel illness characterized by dysbiosis of gut microbiota, damage to the intestinal mucosa, and an imbalance in intestinal immunity (Bu et al., 2025). Immunomodulation and suppression of inflammatory responses are central to the alleviation of UC. The following four studies, all employing DSS-induced mouse models, have investigated the therapeutic effects of *P. pentosaceus* in this context. For instance, EPS produced by *P. pentosaceus* KFT-18 attenuates colonic inflammatory injury by downregulating pro-inflammatory mediators–including IL-6 and IL-1β–and inhibiting NF-κB- and STAT1-associated signaling pathways (Lee J. H. et al., 2022). *P. pentosaceus* M6 attenuates inflammatory responses and alleviates UC by reducing pro-inflammatory cytokines IL-1β and IL-6. Additionally, it restores the intestinal mechanical barrier, limiting exposure of deeper mucosal layers to luminal antigens, bacterial toxins, and other inflammatory stimuli, thereby reducing further mucosal injury (Cao et al., 2025). *P. pentosaceus* CECT 8330 increases the proportion of CD4^+^CD25^+^Foxp3^+^ regulatory T cells (Tregs) in the colonic lamina propria, promoting IL-10 production and contributing to intestinal mucosal immune homeostasis. Additionally, CECT 8330 may alleviate UC by restoring mechanical barrier integrity, increasing SCFA levels, and modulating inflammatory cytokine profiles (Dong et al., 2022). Further studies on *P. pentosaceus* CECT8330 suggest that its ability to promote macrophage polarization from the pro-inflammatory M1 phenotype toward the anti-inflammatory M2 phenotype, reduce M1-derived IL-1β production, and inhibit NF-κB signaling may contribute to its anti-inflammatory effects in UC. Suppression of ROS generation in intestinal epithelial cells represents an additional potential protective mechanism (Hua et al., 2023). Finally, Chen et al. (2020), in a clinical study, reported a significant increase in *P. pentosaceus* abundance within the intestinal mucosal microbiota following administration of a probiotic formulation not containing this species, suggesting probiotic-induced ecosystem remodeling rather than direct supplementation. Its relative abundance was negatively correlated with the UCDAI score, indicating a potential link to symptom alleviation, though a direct causal role remains unestablished (Chen et al., 2020).

#### 4.3.2 Functional gastrointestinal disorders

Functional gastrointestinal disorders (FGIDs) denote a category of gastrointestinal functional diseases characterized by the absence of detectable anatomical abnormalities upon examination, involving disruptions in gut-brain interaction. This includes 33 adult disorders and 20 pediatric ailments. The most common kinds in adults are irritable bowel syndrome (IBS) and functional dyspepsia (FD), whereas the most common in children are Infant colic (IC) and functional constipation (FC) (Zhang et al., 2015; Drossman, 2016). Common pathways of FGIDs include bidirectional failure of the gut-brain axis, dysbiosis of gut microbiota, visceral hypersensitivity, and modifications in mucosal immune function (Black et al., 2020). FGIDs are mostly diagnosed using the Rome criteria; nonetheless, this condition, frequently influenced by psychological factors, can be difficult for patients to accept and may induce feelings of guilt, hence diminishing their propensity to pursue therapy. Probiotics, as a readily incorporated supplementary treatment, may achieve greater acceptance in this environment (Drossman et al., 2009). IBS is categorized into constipation-predominant and diarrhea-predominant forms, both of which are intricately linked to intestinal motility (Shin et al., 2013). *P. pentosaceus* Li05 alleviates IBS-D-associated diarrhea by downregulating 5-HT3BR mRNA expression. As 5-HT3 receptors regulate intestinal motility and secretion, this downregulation may reduce excessive motility and secretion, thereby contributing to symptomatic improvement (Wu et al., 2024). Although the same strain, a separate study proposed that Li05 can alleviate constipation and facilitate fecal excretion. This effect is associated with increased 5-HT production linked to elevated BSH activity in the gut and feces. BSH hydrolyses conjugated bile acids into free bile acids, which activate TGR5 receptors on enterochromaffin cells, upregulating TPH1–the rate-limiting enzyme for 5-HT synthesis–and ultimately promoting 5-HT biosynthesis and secretion. Furthermore, Li05 enhances colonic MUC2 expression, promoting intestinal mucus secretion that lubricates the intestinal lumen and facilitates fecal excretion (Chen et al., 2025). *P. pentosaceus* B49 increased intestinal SCFAs levels in constipated mice. This may contribute to constipation relief by increasing luminal osmotic pressure, improving fecal water content, and potentially activating 5-HT-related pathways to promote intestinal propulsion (Huang et al., 2020).

Infantile colic (IC) is a self-limiting condition characterized by recurrent, prolonged, and unexplained episodes of crying or irritability in otherwise healthy infants. Its pathogenesis remains poorly understood (Zeevenhooven et al., 2018). Existing clinical evidence suggests that a formulation combining *B. longum* KABP042 with *P. pentosaceus* KABP041 can alleviate IC, particularly by reducing crying and fussing time. The underlying effects are thought to involve modulation of gut dysbiosis, enhancement of intestinal barrier integrity, and attenuation of inflammatory responses. The secretion of lactic acid and antimicrobial peptides or bacteriocin-like compounds may help inhibit unfavorable intestinal bacteria and support microbial rebalancing. Nevertheless, as available clinical studies evaluated the combination formulation rather than KABP041 as a single-strain intervention, the strain-specific efficacy of *P. pentosaceus* KABP041 remains to be independently confirmed (Astó et al., 2021; Moreno-Villares et al., 2024).

#### 4.3.3 Colorectal cancer

Recent studies have demonstrated that probiotics can prevent and mitigate adverse responses associated with chemotherapy and immunotherapy (Lei et al., 2025; Yang et al., 2025). During the perioperative phase, the use of probiotics may diminish the occurrence of postoperative infections and gastrointestinal problems (Amitay et al., 2020; Paterson et al., 2025). p8 is an anti-tumor protein that induces G2 phase cell cycle arrest in colorectal cancer cells by downregulating Cyclin B1 and Cdk1, while concurrently upregulating p53 and p21 (An et al., 2019). By introducing the p8 protein gene into *P. pentosaceus SL4*–thereby utilizing this strain as a live biotherapeutic delivery vehicle–protected transport of the p8 protein through the gastrointestinal tract, targeted intestinal release, and sustained local secretion can be achieved (Chung et al., 2021). A further study demonstrated that EPS produced by *P. pentosaceus* E3 exerts anti-colorectal cancer effects by inducing tumor cell apoptosis and disrupting TNF-related tumor growth signaling (Halfawy and Zaghloul, 2025). Further research revealed that *P. pentosaceus* QJ-12 exerts anti-colorectal cancer effects through EPS secretion. EPS inhibits tumor cell proliferation by targeting the spindle assembly checkpoint (SAC) gene, causing cancer cells to arrest at the metaphase of mitosis (Liu et al., 2025). *P. pentosaceus* can also exert anti-cancer effects through the fermentation of dietary substrates. For example, *P. pentosaceus GS4* biohydrogenates dietary linoleic acid (LA) into conjugated linoleic acid (CLA), which subsequently downregulates NF-κB and PI3K–Akt pro-survival signaling, thereby reducing colorectal cancer cell survival and enhancing apoptotic responses (Dubey et al., 2016).

#### 4.3.4 Infectious diarrhea

Despite their antibacterial and antiviral properties, the therapeutic application of probiotics remains limited, and their use in the management of infectious diarrhea is modest (Szajewska et al., 2020). Certain research even indicate that probiotics show no influence on prognosis during treatment (Collinson et al., 2020). *In vitro* studies demonstrate that *P. pentosaceus IM96* directly inhibits the growth of EHEC (Enterohemorrhagic *Escherichia coli*) O157:H7. *In vivo* experiments further indicate that it alleviates EHEC-induced intestinal inflammation, an effect associated with increased intestinal SCFAs production and restoration of both the mechanical and mucosal barriers (Li et al., 2021). *P. pentosaceus SNF15* directly suppresses *E. coli (Escherichia coli) K99 in vitro*, primarily through the production of BLIS and hydrogen peroxide. In infected mice, *SNF15* downregulates jejunal IL-6, IL-1β, and TNF-α at both mRNA and protein levels, restores the intestinal mucus and mechanical barriers, and thereby attenuates inflammation and reduces diarrhea incidence (Su et al., 2025). And *P. pentosaceus SMFM2016-WK1* exerts direct inhibitory effects against the porcine diarrheal pathogen *E. coli in vitro*, possibly through the secretion of BLIS and organic acids. Additionally, this strain may protect intestinal barrier integrity by restoring the mechanical barrier, thereby helping to attenuate host inflammatory responses and reduce diarrhea occurrence (Cho et al., 2025). *P. pentosaceus GT001* reduced the *Salmonella* load in the small intestine, although the study did not provide direct evidence linking this effect to diarrhea prevention or alleviation. Additionally, GT001 enhanced host resistance to infection-associated damage by increasing serum immunoglobulin levels–including IgA, IgG, and IgM–and strengthening antioxidant defenses (Bumbie et al., 2024).

#### 4.3.5 Summary

Overall, the shared mechanisms of *P. pentosaceus* in gastrointestinal diseases include suppression of excessive inflammatory responses, enhancement of intestinal epithelial and mucus barrier integrity, modulation of gut microbiota composition and metabolic outputs, and regulation of host immune homeostasis. In the context of colorectal cancer, additional mechanisms encompass inhibition of tumor cell proliferation, induction of apoptosis, and suppression of pro-survival signaling pathways, mediated either directly or via metabolic products. Nevertheless, the current evidence base remains insufficient to support robust clinical translation. Most mechanistic findings derive from *in vitro* experiments or animal models, while rigorously designed human clinical trials remain scarce. Where clinical evidence exists, it is frequently based on multi-strain formulations, making it difficult to determine the specific contribution of *P. pentosaceus*. Furthermore, many proposed mechanisms are inferred from synthesized prior findings rather than confirmed through causal intervention studies.

### 4.4 Diabetes

Probiotic therapy for diabetes has been validated in multiple randomized controlled trials (RCTs), including both type 1 and type 2 diabetes (Rittiphairoj et al., 2021; Baroni et al., 2024). Its primary mechanisms include inhibiting digestive enzyme activity, attenuating systemic inflammation-induced insulin resistance, mitigating oxidative stress- and inflammation-mediated pancreatic islet damage, and exerting regulatory effects through microbial metabolites such as SCFAs (Ağagündüz et al., 2025).

#### 4.4.1 α-amylase and α-glucosidase

α-Amylase, secreted primarily by the salivary glands and pancreas, hydrolyses internal α-1,4 glycosidic bonds in starch. In the oral cavity, salivary α-amylase initiates starch digestion, producing shorter polysaccharides and dextrins. Upon entering the small intestine, partially digested starch and longer-chain dextrins are further hydrolyzed by pancreatic α-amylase into short-chain carbohydrates, including oligosaccharides and short-chain dextrins (Kaur et al., 2021). Subsequently, α-glucosidase–localized primarily at the brush border of small intestinal epithelial cells–catalyzes the conversion of these α-amylase-derived short-chain carbohydrates into absorbable monosaccharides via hydrolysis of their α-glycosidic bonds, thereby contributing to the elevation of postprandial blood glucose levels (Dirir et al., 2022). An *in vitro* study shown that *P. pentosaceus KI62* reduced blood glucose levels by inhibiting α-glucosidase and α-amylase, achieving inhibition rates of 98.59% ± 0.52% and 94.86% ± 3.30%, respectively (Kim S. et al., 2021).

#### 4.4.2 GLP-1

GLP-1 is a 31-amino acid peptide classified as an intestinal hypoglycemic hormone, mostly produced by intestinal L cells, and is crucial in regulating blood glucose levels (Wang et al., 2016). The regulatory functions of GLP-1 include enhancing insulin sensitivity in peripheral tissues, stimulating pancreatic β-cell proliferation, and modulating the vagus nerve and central nervous system to suppress food consumption (Drucker, 2018; Pegah et al., 2021). *P. pentosaceus MF000957*, derived from camel milk, ferments milk proteins to produce bioactive peptides with DPP-IV inhibitory potential. By inhibiting DPP-IV–an enzyme capable of rapidly degrading GLP-1–these peptides may prolong GLP-1 activity and thereby indirectly contribute to glycemic regulation (Mudgil et al., 2024). SCFAs–particularly butyrate–can also promote GLP-1 release (Yadav et al., 2013; Wang et al., 2025). Given that certain *P. pentosaceus* strains have been shown to increase intestinal SCFAs levels, this may represent a potential blood glucose-lowering mechanism; however, direct experimental evidence confirming this hypothesis remains lacking.

#### 4.4.3 Inflammation-induced insulin resistance

Research indicates that serum LPS concentrations are elevated in individuals with diabetes, and LPS has been shown to be associated with systemic inflammation (Yuan et al., 2021). Systemic inflammation may interfere with insulin binding to its receptors, thereby contributing to dysregulated blood glucose levels (Ding et al., 2021). Research on *P. pentosaceus E24-168* revealed that obesity-induced insulin resistance may be linked to obesity-generated inflammatory factors and ROS, which interfere with insulin signaling and thereby contribute to insulin resistance (Habib et al., 2026). Animal studies have confirmed that *P. pentosaceus* PP04 can ameliorate high-fat diet-induced insulin resistance, an effect potentially associated with the attenuation of systemic inflammatory responses (Wang et al., 2025).

#### 4.4.4 Summary

Overall, the glucose-lowering effects of *P. pentosaceus* are unlikely to depend on a single mechanism. Rather, they appear to involve multiple complementary pathways, including inhibition of digestive enzymes to reduce carbohydrate absorption and postprandial glycaemic excursions, attenuation of chronic inflammation to improve insulin sensitivity, and modulation of insulin secretion and incretin-related pathways. Accordingly, *P. pentosaceus* is more appropriately regarded as an adjunctive strategy for metabolic regulation than as a standalone therapy capable of replacing established glucose-lowering medications. Nevertheless, clinical evidence in this area remains limited, and the translational potential of these effects remains largely theoretical, pending validation through well-designed clinical studies.

### 4.5 Respiratory system diseases

Multiple studies have shown the correlation between gut microbiota and the respiratory disorders, including chronic obstructive pulmonary disease (COPD), asthma, cystic fibrosis (CF), lung cancer, and respiratory infections (Chunxi et al., 2020). Its mechanism involves bidirectional immunological interactions mediated through the gut–lung axis (GLA) (Eladham et al., 2024). The gastrointestinal tract and pulmonary system both constitute components of the mucosal immune system, enabling immune cells to migrate between these sites via shared pathways (Shahbazi et al., 2020). Gut microbiota dysbiosis impairs intestinal barrier function and stimulates local pro-inflammatory mediator production, promoting the recruitment of immune cells, including neutrophils and T lymphocytes (Birring et al., 2003; Hu et al., 2025). These activated immune cells subsequently enter the systemic circulation and traffic to the lungs, where they trigger pulmonary inflammatory responses. This aberrant migratory pattern is referred to as error homing, a phenomenon in which immune cells are misdirected to inappropriate anatomical sites, often driven by a dysregulated internal milieu (Adams and Eksteen, 2006; Mateer et al., 2015). A study has confirmed that intestinal inflammation disrupts the epithelial barrier, facilitating systemic dissemination of gut-derived inflammatory mediators. The resulting chronic inflammatory milieu upregulates aberrant pulmonary homing signals–including CCL25 and MAdCAM-1–driving the ectopic recruitment of intestinal inflammation-primed α4β7^+^ and CCR9^+^ CD4^+^ T cells to lung tissue. This process exacerbates pulmonary inflammation, providing a mechanistic illustration of the gut–lung immune axis (Eladham et al., 2025). Furthermore, compromised intestinal barrier integrity may facilitate the direct translocation of intestinal metabolites into the circulatory system, subsequently impacting pulmonary function (Deitch, 2010). An experimental sTBI mouse model demonstrated that approximately 49.69% of the lung microbiota at day 7 post-injury was predicted to originate from the gut. This translocation may be attributed to sTBI-induced intestinal barrier impairment and reduced antimicrobial peptide secretion by Paneth cells, thereby facilitating the hematogenous dissemination of gut-derived bacteria to the lungs (Yang et al., 2022). Although direct evidence that *P. pentosaceus* reduces aberrant pulmonary immune cell homing or gut-derived bacterial translocation to the lungs is currently lacking, mouse studies indicate that certain strains can alleviate pulmonary inflammation via gut–lung axis-related mechanisms. *P. pentosaceus SMM914* attenuates COPD progression by inhibiting Hspa1a- and TLR4-associated signaling pathways (Liu Y. et al., 2024), whereas *P. pentosaceus MIANGUAN2* mitigates influenza-induced lung injury through downregulation of TNF- and NF-κB-mediated inflammatory responses (Chen et al., 2024). Both effects are associated with increased enrichment of SCFA-producing gut bacteria, particularly Lachnospiraceae, suggesting that these strains may indirectly modulate pulmonary inflammation by reshaping the gut microbiota. Additionally, *P. pentosaceus* can also indirectly alleviate lung injury. *P. pentosaceus ON81A* ferments germinated *Rhynchosia nulubilis* to produce GR-ON81A, which is enriched in polyphenolic antioxidant compounds and exhibits strong DPPH free radical-scavenging activity. In an *in vitro* particulate matter-induced cell injury model, GR-ON81A showed a protective effect by attenuating alveolar epithelial cell death (Lee and Park, 2021).

#### 4.5.1 Summary

Overall, existing evidence suggests that the effects of *P. pentosaceus* on respiratory diseases are primarily mediated through indirect gut–lung axis-related mechanisms, rather than through direct pulmonary colonization or direct pathogen clearance. However, the current evidence remains largely limited to preclinical studies, and the mechanistic connection between gut microbial modulation and pulmonary protection has not yet been fully clarified. In particular, there is still no conclusive evidence demonstrating that *P. pentosaceus* directly suppresses aberrant immune-cell homing, reduces gut-derived bacterial translocation, or regulates the transport of specific gut-derived metabolites to the lungs.

### 4.6 Neurological and mental disorders

Recent research have investigated the application of probiotics in enhancing neurological and psychiatric conditions, such as depression (Schaub et al., 2022), anxiety (Asad et al., 2025), autism spectrum disorder (ASD) (Narula Khanna et al., 2025), multiple sclerosis (MS) (Straus Farber et al., 2024), Alzheimer’s disease (AD) (Ding et al., 2021), Parkinson’s disease (PD) (Leta et al., 2025), hepatic encephalopathy (HE) (Manothiya et al., 2025), and stroke (Ademoyegun et al., 2025). The gut–brain axis is a complex regulatory network encompassing neurological, immunological, metabolic, and endocrine pathways. Compromised intestinal barrier function facilitates the translocation of inflammatory mediators and gut metabolites to the central nervous system via the bloodstream, thereby significantly contributing to disease development (Cryan et al., 2019).

#### 4.6.1 Parkinson’s disease (PD)

Parkinson’s disease (PD) is a neurodegenerative disorder primarily characterized by motor impairment, frequently accompanied by non-motor symptoms including autonomic and enteric nervous system dysfunction, manifesting as slowed intestinal transit, constipation, and sensory disturbances (Martin et al., 2018). *P. pentosaceus WMU002* attenuates neuronal oxidative damage by modulating the Keap1/Nrf2 antioxidant signaling pathway. It also increases brain GABA levels, though the precise mechanisms underlying this effect remain to be fully elucidated (Pan et al., 2022). *P. pentosaceus ENM104* lacks the canonical *gadA*/*gadB* genes required for efficient GABA biosynthesis, carrying only *gadC*–encoding a glutamate/GABA antiporter–and therefore exhibits limited GABA-producing capacity (Kompramool et al., 2024). GABA is an inhibitory neurotransmitter whose levels are significantly reduced in the central nervous system of patients with Parkinson’s disease, with lower levels negatively correlated with the severity of muscle rigidity and tremor. Accordingly, *P. pentosaceus*–by virtue of its capacity to promote GABA production–holds potential therapeutic value in alleviating PD-associated symptoms (van Nuland et al., 2020; Song et al., 2021).

#### 4.6.2 Anxiety

Anxiety disorders are closely associated with dysregulation of the brain–gut–microbiome (BGM) axis (Martin et al., 2018). Probiotics may alleviate anxiety by upregulating brain-derived neurotrophic factor (BDNF) expression, and promoting the synthesis of key neurotransmitters including GABA and 5-HT. They may also attenuate inflammation-related hypothalamic–pituitary–adrenal (HPA) axis dysregulation, thereby contributing to improved emotional regulation (Zhang et al., 2025). A study involving *P. pentosaceus E24-168* suggests that a high-fat diet may disrupt hypothalamic–pituitary–adrenal (HPA) axis homeostasis–thereby inducing anxiety-like behavior in mice–by upregulating pro-inflammatory mediators including IL-1β, NLRP3, and Caspase-1. While E24-168 appears to alleviate these anxiety-like behaviors, with particularly pronounced effects when combined with exercise intervention, the specific underlying mechanisms have not yet been directly verified (Habib et al., 2026).

#### 4.6.3 Alzheimer’s disease (AD)

Alzheimer’s disease (AD) is a chronic, progressive neurodegenerative disorder characterized primarily by the extracellular accumulation of β-amyloid–particularly Aβ42–forming amyloid plaques, and the abnormal intracellular hyperphosphorylation of Tau protein, giving rise to neurofibrillary tangles (NFTs). These pathological changes promote mitochondrial dysfunction, oxidative stress, and microglia-mediated neuroinflammation, thereby activating neuronal necrotic and apoptotic pathways, ultimately leading to synaptic degeneration, neuronal loss, and progressive cognitive decline (Goel et al., 2022). An *in vitro* study demonstrated that the culture supernatant of *P. pentosaceus* WB3812 enhances Nrf2-mediated antioxidant defenses and reduces ROS accumulation, thereby lowering the Bax/Bcl-2 ratio, stabilizing the mitochondrial apoptotic pathway, and inhibiting caspase-9/caspase-3 cascade activation, ultimately attenuating H_2_O_2_-induced apoptosis in neuron-like cells. However, since this mechanism was validated only *in vitro*, its applicability to Alzheimer’s disease still requires further verification (Lee et al., 2025).

#### 4.6.4 Summary

Overall, the potential benefits of *P. pentosaceus* in neurological and psychiatric disorders are primarily mediated through the gut–brain axis rather than through direct action on the central nervous system. These findings suggest that *P. pentosaceus* may function as an auxiliary modulator of neuroimmune and neuroendocrine homeostasis, particularly by attenuating peripheral inflammatory signals that influence central nervous system function. However, as current evidence derives exclusively from cell-based experiments and animal models, a definitive causal relationship between probiotic intervention and improved neurological outcomes has yet to be established.

### 4.7 Other diseases

Beyond the conditions discussed above, *P. pentosaceus* demonstrates potential applicability in several other disease contexts; however, given the limited number of available studies, these are collectively presented here.

In a treadmill-induced fatigue mouse model, *P. pentosaceus* YF01 enhanced antioxidant capacity and improved intramuscular energy metabolism by upregulating the mRNA expression of MyHC I, SIRT1, and PGC-1α in muscle tissue, as well as antioxidant enzymes SOD1, SOD2, and CAT in skeletal muscle and liver tissues, ultimately improving exercise endurance and anti-fatigue performance (Yang et al., 2024). In an *in vitro* wound-healing model using L929 mouse fibroblast cells, both live cells and lysates of *P. pentosaceus* AF2 promoted scratch closure, with live bacterial treatment demonstrating a superior effect. These findings suggest that AF2 may accelerate wound repair by enhancing fibroblast migration or releasing bioactive metabolites that support tissue regeneration (Ugras et al., 2024). *P. pentosaceus* can mitigate certain forms of poisoning; specifically, *P. pentosaceus* MT323062 facilitates the excretion of cadmium from the intestines, thus decreasing cadmium toxicity (Hameed et al., 2025). In a diquat-induced intestinal oxidative stress mouse model, *P. pentosaceus* ZJUAF-4 alleviated intestinal injury by restoring jejunal villus and microvillus integrity, enhancing SOD-mediated antioxidant defense, and activating the Nrf2- related antioxidant signaling pathway (Hao et al., 2021). *In vitro* assays using artificial saliva models demonstrated that *P. pentosaceus* 732 maintained good survival under simulated oral conditions. Furthermore, susceptibility testing revealed that most commercially available medications and oral care products exerted little or no inhibitory effect on its growth. These findings suggest that *P. pentosaceus* 732 may hold potential as an oral probiotic candidate for oral health applications (Choi et al., 2025).

In a 4T1 orthotopic breast cancer mouse model, oral administration of *P. pentosaceus PP06* in combination with intravenous doxorubicin (DOX) enhanced local anti-tumor immune responses. The combined treatment upregulated pro-inflammatory and immune-activating cytokines, including TNF-α, IFN-γ, and IFN-β, which are closely associated with CD8^+^ T-cell activation, macrophage function, and amplification of anti-tumor immunity, suggesting potential as an adjuvant probiotic in chemotherapy settings (Ye et al., 2024). In an *in vitro* endothelial inflammation model using LPS-stimulated human umbilical vein endothelial cells (HUVECs), Yak-Kong soybeans fermented by *P. pentosaceus AOA2017* reduced LPS-induced intracellular ROS accumulation by increasing the content of bioactive phenolic acids, particularly *p*-coumaric acid. This was accompanied by downregulation of vascular cell adhesion molecule-1 (VCAM-1) expression, thereby reducing THP-1 monocyte adhesion to endothelial cells–a key early step in the inflammatory cascade underlying atherosclerosis (Kim et al., 2019). In a 5-fluorouracil (5-FU)-induced intestinal mucositis mouse model, oral administration of *P. pentosaceus PP34* enhanced systemic antioxidant capacity, as reflected by increased T-SOD and GSH-Px levels. Additionally, PP34 attenuated jejunal inflammation by downregulating CXCL-9, CXCL-5, and NLRP3 expression, thereby alleviating the 5-FU-induced inflammatory response (He et al., 2025).

Extra-intestinal translocation of *P. pentosaceus* may contribute to disease development. A case of *P. pentosaceus*-associated endocarditis was reported in Greece in 2024. The authors postulated that the patient’s underlying liver cirrhosis led to chronic intestinal venous congestion, resulting in intestinal mucosal malnutrition and compromised intestinal barrier function, which facilitated *P. pentosaceus* translocation into the systemic circulation and ultimately precipitated endocarditis (Mantzios et al., 2024). Finally, clinical observations have revealed that the prevalence of *P. pentosaceus* in semen is positively associated with sperm concentration and negatively correlated with body mass index (BMI), suggesting that obesity may elevate the risk of infertility (Yao et al., 2025).

## 5 Discussion

### 5.1 Experimental efficacy versus clinical efficacy

Although numerous studies have confirmed the beneficial effects of *P. pentosaceus in vitro* and in animal models, these systems cannot fully replicate the complexity of the human gut ecosystem, including microbial competition, host diet, immune status, medication use, and disease heterogeneity–all of which differ substantially from the clinical environment. The therapeutic effects observed in experimental models therefore represent promising directions for future investigation, but extensive mechanistic validation, along with rigorous assessment of strain safety and stability, remains necessary before clinical translation can be achieved.

### 5.2 Limitations of current evidence

Despite the promising probiotic properties and disease-modifying potential of *P. pentosaceus*, several limitations remain. First, most existing evidence is derived from *in vitro* experiments and animal models, while well-designed and well-interpreted human studies are scarce. Furthermore, some studies have used multiple probiotic formulations, making it difficult to pinpoint the specific role of *P. pentosaceus*. Significant heterogeneity exists across different studies in terms of strain origin, dosage, duration of intervention, disease models, and outcome metrics, limiting direct comparisons between studies and hindering the development of standardized therapeutic protocols. Many proposed mechanisms–such as SCFA-mediated regulation, bile acid metabolism, intestinal barrier repair, immune regulation, and antioxidant signaling–are primarily based on correlational studies rather than causal intervention experiments. Therefore, some mechanistic connections remain at the inferential stage and require further validation using loss-of-function targeting, metabolite supplementation, receptor blockade, or germ-free/transplantation models. Safety is essential for assessing clinical translational potential, but research often lacks comprehensive safety evaluation. For example, while some strains may not exhibit hemolytic activity and lack significant virulence or transferable antibiotic resistance genes, most safety studies are short-term with small sample sizes or lack systematic follow-up. Furthermore, evidence in susceptible populations such as infants, the elderly, and immunocompromised patients remains insufficient. Current research primarily focuses on MASLD, UC, obesity-related hypercholesterolaemia, and DM, while research on other conditions remains limited or in its early stages, particularly in the areas of respiratory, neurological, and psychiatric diseases. Finally, because the biological effects of *P. pentosaceus* are highly strain-specific, results from single-strain studies cannot be representative of the entire species. Future research should prioritize standardized strain identification, dose-response assessment, direct validation of causal mechanisms, long-term oral safety assessment, and single-strain randomized controlled trials.

## 6 Conclusion

This review integrates current evidence on the probiotic properties, safety profile, and multi-system therapeutic applications of *P. pentosaceus*, comparing strain-specific mechanisms and grading studies according to evidence type. Current evidence suggests that *P. pentosaceus* may exert beneficial effects through multiple interrelated mechanisms, including intestinal barrier enhancement, antimicrobial metabolite production, antioxidant modulation, immunomodulation, and regulation of gut-derived metabolites such as SCFAs, bile acids, and indole derivatives. However, most evidence remains preclinical, and clinical translation is constrained by strain heterogeneity, limited single-strain trials, short follow-up periods, and incomplete safety assessments. Future research should prioritize standardized strain characterization, causal mechanism validation, dose optimization, long-term safety evaluation, and well-designed single-strain randomized controlled trials. Overall, *P. pentosaceus* represents a promising but insufficiently validated probiotic candidate with potential as an adjunctive therapy for systemic diseases. Nevertheless, its clinical application requires cautious advancement through rigorous experimental validation and evidence-based clinical investigation.
